# Parkinson’s associated protein DJ-1 regulates intercellular communication via extracellular vesicles in oxidative stress

**DOI:** 10.1038/s41420-025-02845-7

**Published:** 2025-11-21

**Authors:** Thomas Page, Clara Alice Musi, Saskia E. Bakker, David R. Jenkins, Eric J. Hill, Tiziana Borsello, Ivana Milic, Andrew Devitt, Mariaelena Repici

**Affiliations:** 1 Aston Institute for Membrane Excellence, School of Biosciences, College of Health and Life Sciences, Aston University, Birmingham, UK; 2 Department of Pharmacological and Biomolecular Sciences, Università degli Studi di Milano, Milano, Italy; 3 Mario Negri Institute for Pharmacological Research – IRCCS, Milano, Italy; 4 Research Technology Platform, University of Warwick, Coventry, UK; 5 Department of Chemistry, Loughborough University, Epinal Way, Loughborough, UK

**Keywords:** Parkinson's disease, Mechanisms of disease

## Abstract

Mutations in DJ-1 cause autosomal recessive Parkinson’s disease (PD). Several functions have been attributed to DJ-1, including a key role in the protection from oxidative stress. However, how this protein contributes to PD pathogenesis is still unclear. Recently, DJ-1 has been identified at higher concentrations in extracellular vesicles (EV) from biological fluids of PD patients, providing a link between EV and a protein associated with PD. In this study, EV were purified from the medium of control and rotenone-treated wild-type and DJ-1 KO differentiated SH-SY5Y cells. EV quantity was assessed using flow cytometry, and their proteomic cargo was analysed via mass spectrometry. We identified an altered EV response to rotenone in DJ-1 KO cells compared to wild-type. Mass spectrometry analysis identified 116 proteins with significantly altered abundance between the two genotypes, indicating a role for DJ-1 in modulating EV cargo under oxidative stress conditions. Label-free identification of oxidative modifications indicated that DJ-1 clearly influences the oxidative profile of EV proteins. Additionally, we showed that DJ-1 KO alters the ability of the secretome to stimulate macrophage migration, suggesting functional consequences of DJ-1 deficiency in secretome-mediated responses to oxidative stress. The altered EV response to rotenone was confirmed in iPSC-derived neurons lacking DJ-1 compared to isogenic controls. Our results reveal a distinct role for DJ-1 in regulating intercellular communication under oxidative stress, highlighting a novel EV-mediated function of DJ-1 that may contribute to Parkinson’s disease pathogenesis.

## Introduction

Mutations in DJ-1, encoded by the *PARK7* gene, cause autosomal recessive Parkinson’s disease (PD) [1, 2]. Despite a huge number of studies to elucidate the exact role of DJ-1 in the pathogenesis of PD, the key molecular mechanisms are not yet clear. While DJ-1 is known to play a role in the protection against oxidative stress [3], it is also implicated in mitochondrial homeostasis, regulation of apoptosis and autophagy [4], dopamine synthesis and reuptake [5], and regulation of the immune system [6, 7]. Recently, DJ-1 has been identified at higher concentration in extracellular vesicles (EV) [8–10] from biological fluids of PD patients, providing a link between EV and a protein associated with PD.

EV are small bilipid layer-enclosed vesicles, produced by a wide variety of cells and secreted into the extracellular environment, with a key role in intercellular communication [11]. They contain a broad spectrum of proteins, lipids, and nucleic acids that are cell and context-specific [12]. In the CNS, EV can be secreted by all types of brain cells [13] and play a role in synaptic function, synaptic plasticity, and myelin production, neuronal development, and maturation [14, 15]. Interestingly, increasing literature has reported roles of EV in the occurrence and progression of neurodegenerative disorders, including PD [16–18]. The increased presence of DJ-1 in EV derived from PD patients is intriguing for two reasons: first, exosomal DJ-1 could represent a viable PD biomarker [19, 20] and second, it could inform new molecular mechanisms responsible for PD pathogenesis [21, 22].

Here, we investigated the role of DJ-1 in EV-mediated intercellular communication and assessed the consequences of DJ-1 absence in such communication in differentiated SH-SY5Y cells upon oxidative stress. Using mass spectrometry, we identified a distinct proteomic signature in EV derived from DJ-1-deficient cells compared to those from wild-type cells. Furthermore, we demonstrated that the secretome from DJ-1 KO cells exposed to oxidative stress exhibits functional differences compared to wild-type cells in its impact on immune cell migration. Notably, we observed that the EV response to oxidative stress in DJ-1 knockout iPSC-derived neurons differs from that in wild-type cells, further validating the findings from our in vitro model.

## Results

### DJ-1 KO affects EV concentration in differentiated SH-SY5Y cells

**Fig. 1:**
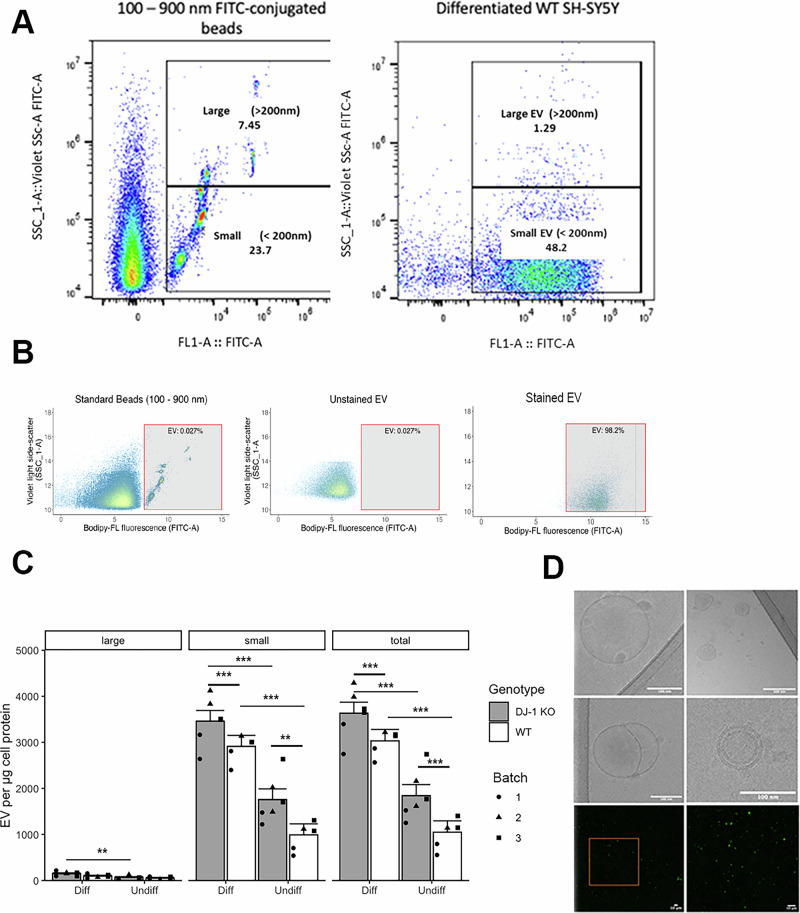
Both differentiation and DJ-1 KO increase the amount of EV in SH-SY5Y cells. **A** Flow cytometry gating strategy (Beckman Coulter CytoFLEX S flow cytometer). Left dot plot: 1:1 mix of Megamix-Plus SSC and Megamix-Plus FSC beads (BioCytex, UK) ranging from 100 nm to 900 nm diameter was used to determine subsequent size gating. Two distinct populations (small particles, <200 nm, and large particles, >200 nm) are defined. Right dot plot: gating for the identification of small and large EV present in the medium of differentiated SH-SY5Y cells. **B** Comparison of the flow cytometry data obtained for standard sized green fluorescent beads, unstained EV, and Bodipy-FL stained EV, showing how correct gating of EV was achieved. *Y* axis = violet light side scatter. *X* axis = Bodipy fluorescence. EV gate is indicated by red bordered rectangle. Local density of points represented by colour with blue = lowest and red = highest density. All data were generated on Beckman Coulter CytoFLEX S flow cytometer. Fluorescence data were transformed using a biexponential function. **C** Number of EV per µg of protein detected by flow cytometry on Beckman Coulter CytoFLEX S flow cytometer. EV counts are shown as a total (right panel), small (<200 nm diameter, middle panel), large (>200 nm diameter, left panel). Columns represent least square means calculated using emmeans R package, from data modelled as a linear mixed effects model of the form: Response variable ~Genotype*Differentiation state + (1│Batch) using lme R package. n = 9. Differentiation increased the number of EV present in the medium (2.9-fold-change in wild-type cells, p < 0.0001; 1.96 fold-change in DJ-1KO cells, p < 0.0001, Tukey HSD for multiple comparisons). When comparing differentiated cells only, an increase of EV in DJ-1 KO cells compared to wild-type was observed (1.18-fold change, *p* = 0.0083, Tukey HSD for multiple comparisons). Error bars: standard error of the emmeans least square mean calculation. (* = p < 0.05, ** = p < 0.005, *** = p < 0.001). **D** Upper panels: Cryo-EM images of the variety of EV shapes obtained from differentiated SH-SY5Y cells. Scale bar = 100 nm. Lower panel: Confocal microscopy images of EV isolated from differentiated SH-SY5Y cells stained with MemglowTM 488. Left image: full image at native resolution, right image: zoomed in selection of the left image. Clear independent fluorescent objects were observed within the size range expected of EV (<1 µm) with little to no background noise. Scale bar = 10 µm.

To investigate DJ-1 role in intercellular communication, we first characterized the EV population produced by wild-type and DJ-1 KO differentiated SH-SY5Y cells in control conditions. To date, studies on SH-SY5Y EV have been narrowly focussed on exosomes [23–25], identified by the standard exosome markers described in MISEV 2014 [26]. We here took a wider approach and interrogated EV based on size, considering EV below 200 nm diameter as small EV and those above 200 nm as large, and making no assumptions of biosynthetic origin as recommended in MISEV 2023 [27]. EV present in the cell medium were stained with BODIPY FL-SE and detected by flow cytometry (Fig. 1A, B). To account for variations in the number of donor cells in each condition, we normalised EV per µg of protein lysates. Our results show an increase in the number of total EV upon differentiation both in wild-type cells and DJ-1KO cells, primarily driven by changes in small EV (Fig. 1C). Interestingly, DJ-1 KO cells showed higher small EV counts than wild-type cells (1.18-fold change) irrespective of differentiation state. Cryo-EM studies confirmed structural details and morphological features of EV obtained from SH-SY5Y cells (Fig. 1D).

### EV response to rotenone is different in WT and DJ-1 KO differentiated SH-SY5Y

Given the critical role of oxidative stress in PD pathogenesis [28] and the well-established function of DJ-1 in the cellular response to oxidative stress [29, 30], we next examined whether the EV differences observed under control conditions were maintained during oxidative stress. We first optimized the rotenone concentration that minimally affects differentiated SH-SY5Y cells by analysing parameters related to different aspects of cell death: total nuclei, necrotic nuclei percentage, nuclei circularity. An increase in circularity indicates enhanced apoptosis, as nuclear shrinkage and condensation, hallmark features of apoptosis, lead to a more uniform, circular shape [31]. No differences were found in the percentage of necrotic nuclei upon rotenone treatment for both genotypes (Supplementary Fig. 2A), suggesting that all tested treatments of rotenone are not inducing primary necrosis, and that apoptotic events are primarily non-necrotic and largely early stage. Nuclear circularity slightly increased upon rotenone treatment in both WT and DJ-1 KO genotypes at 5, 10, 25, and 50 nM rotenone compared to their respective control (Supplementary Fig. 2B), indicating that the rotenone concentrations used were only causing minimal apoptosis with no further increase in DJ-1 KO cells compared to wild-type cells.

**Fig. 2:**
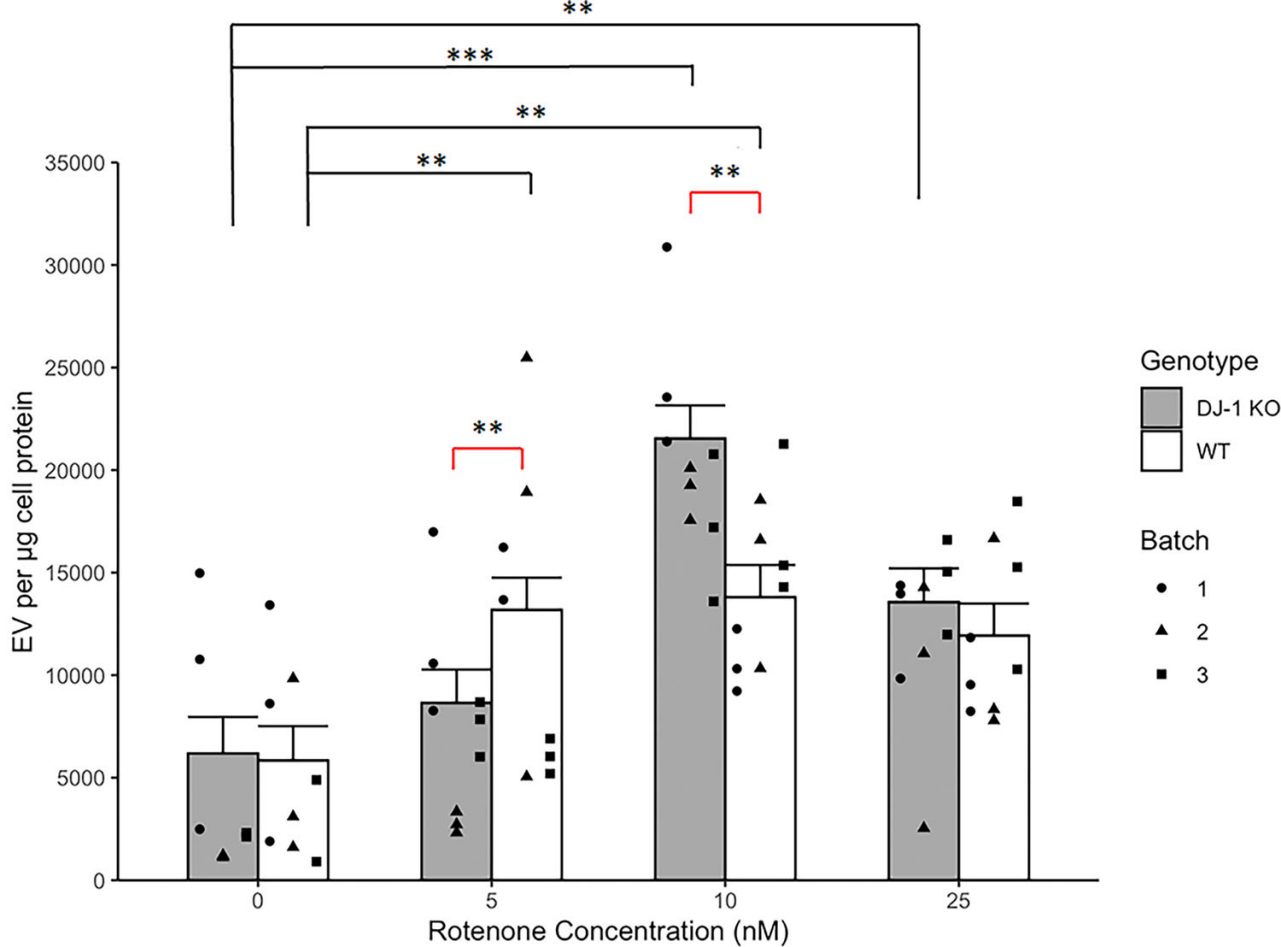
Rotenone treatment results in a genotype dependent increase in small EV in differentiated SH-SY5Y cells. Amount of small EV detected by flow cytometry from differentiated WT and DJ-1 KO SH-SY5Y cells with or without rotenone treatment. EV were detected on Cytoflex S system, EV size was assessed by violet-light side scatter. EV events were separated from noise by the fluorescence of Bodipy-FL-SE staining. Analyses of small EV number were carried out using the emmeans package in R on a mixed effect model of the form: EV number ~ Rotenone Concentration ∗ Genotype + (1|batch) using lme R package. *n* = 9. Column height = least square mean calculated by the emmeans R package. EV from WT cells were significantly different from control at treatments of 5 (p = 0.006) and 10 (p = 0.003) nM rotenone, increasing by factors of 2.25 and 2.36, respectively. However, in DJ-1 KO, small EV significantly increased at treatments of 10 (p < 0.0001) and 25 (p = 0.006) nM rotenone by factors of 3.48 and 2.19 respectively. Furthermore, at 5 nM rotenone significantly higher numbers of small EV were detected in WT cells, 1.52 times higher than the amount detected in DJ-1 KO EV (p = 0.05), whereas this effect is reversed at 10 nM rotenone treatment with more EV being detected from DJ-1 KO cells compared to WT (1.56 fold). Error bars = standard error. *p < 0.05; **p < 0.01, and ***p < 0.001. Tukey HSD for multiple comparisons.

As previously observed in control conditions (Fig. 1), the proportion of the EV population designated as large (>200 nm diameter) upon rotenone treatment was substantially lower than the small population and no significant differences were observed between genotypes following rotenone treatment (Supplementary Fig. 3), suggesting that the cellular response to rotenone in our model is primarily reflected in the small EV (<200 nm). There was no significant difference in small EV between the two genotypes under baseline conditions (no rotenone) (Fig. 2). This contrasts with the data in Fig. 1, likely due to the different EV collection times, 48 h in Fig. 1 versus 24 h in Fig. 2. Interestingly, the EV response to the range of rotenone concentrations was different in WT and DJ-1 KO cells (Fig. 2). Our data show an increase of 2.25-fold at 5 nM rotenone (p = 0.006) and 2.36-fold at 10 nM rotenone (p = 0.003) compared to control conditions in small EV from WT cells. However, in DJ-1 KO, small EV significantly increased at 10 nM rotenone (3.48-fold, p < 0.0001) and 25 nM rotenone (2.19-fold, p = 0.006) (Fig. 2) compared to their respective control. At 5 nM rotenone, WT cells showed a significantly higher number of small EV than DJ-1 KO cells (1.52-fold). However, this trend reversed at 10 nM rotenone, where DJ-1 KO cells produced 1.56-fold more EV compared to WT cells (Fig. 2). Since 10 nM rotenone was the only concentration that produced significant differences both within each genotype (compared to the control) and between the genotypes, it was selected for further investigations.

### 10 nM rotenone treatment results in genotype dependent changes in mitochondria morphology

**Fig. 3:**
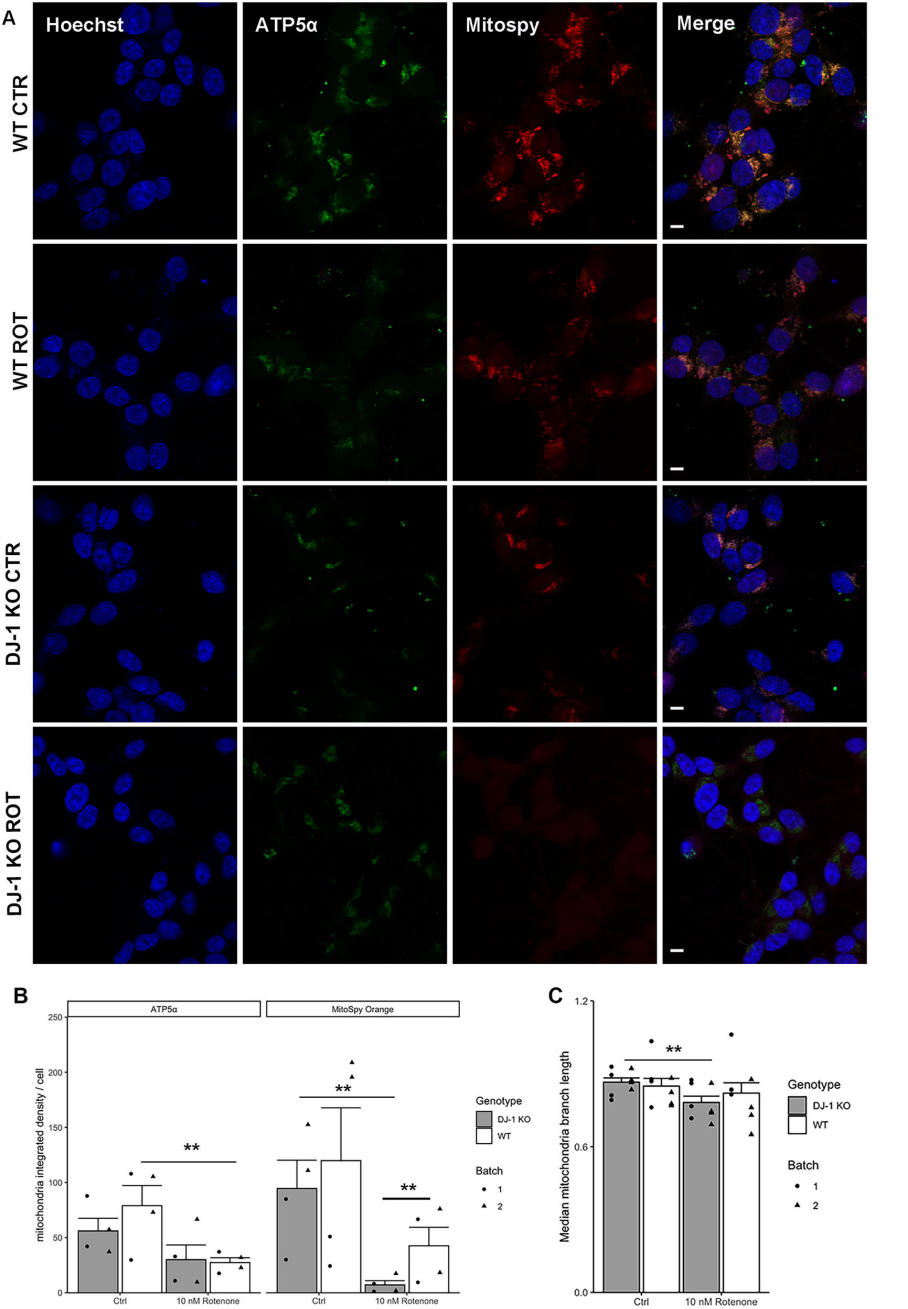
Rotenone treatment causes genotype dependent changes in mitochondria morphology. **A** Confocal microscopy of ATP5-alpha and Mitospy orange labelled mitochondria in differentiated WT and DJ-1 KO SH-SY5Y with or without rotenone treatment captured on a Leica SP8 confocal microscope. Scale bar = 6 µm. Quantification of mitochondria morphology as integrated density (**B**) and average mitochondrial branch length (**C**). Significant differences were determined via the emmeans R package, employing Dunnet’s test. Column height = least square mean. Error bars: standard error of the emmeans least square mean calculation. ATP5-alpha immunofluorescence integrated density per cell decreased 2.88-fold in WT differentiated SH-SY5Y when treated with rotenone (p = 0.038). Mitospy orange integrated density per cell decreased 2.8-fold in WT (p = 0.05) and 10.6-fold in DJ-1 KO (p = 0.037) cells upon rotenone treatment. Rotenone treatment reduced the maximum mitochondria branch length (1.2 fold) of DJ-1 KO cells but not wild-type cells (p = 0.0248), n = 4.

As a potent inhibitor of mitochondrial complex I, the effect of 10 nM rotenone on the morphology of mitochondria in differentiated SH-SY5Y cells was also investigated. Mitochondrial stress induced by the toxin was studied by using a double readout of mitochondria healthy state: the polarised (healthy) mitochondria selective dye Mitospy orange and the immunofluorescent staining of the mitochondrial protein ATP5-alpha, known to undergo changes upon oxidative stress [32]. Confocal microscopy of both ATP5-alpha and Mitospy orange revealed strong and clear staining of mitochondrial structures in control conditions for WT and DJ-1 KO differentiated SH-SY5Y with a decrease in fluorescence observed upon rotenone treatment in both genotypes (Fig. 3A). To confirm these qualitative data, we quantified mitochondria staining in both ATP5-alpha and Mitospy orange labelled cells (Fig. 3B) and we analysed the average mitochondrial branch length upon Mitospy orange staining, as this staining was stronger than ATP5-alpha (Fig. 3C). ATP5-alpha immunofluorescence integrated density per cell decreased 2.88-fold in WT differentiated SH-SY5Y when treated with rotenone (p = 0.038), while Mitospy integrated density per cell decreased 2.8-fold in WT (p = 0.05) and 10.6-fold (p = 0.037) in DJ-1 KO cells upon rotenone treatment (Fig. 3B), thus confirming a higher effect of 10 nM rotenone on mitochondria in cells lacking DJ-1. Upon rotenone treatment, DJ-1 KO cells exhibited a 3.8-fold decrease in integrated density per cell compared to WT cells (p = 0.01), indicating a mitochondrial phenotype that is DJ-1 dependent (Fig. 3B). Lastly, network branch analysis of Mitospy labelled mitochondria showed that rotenone treatment reduced by 1.2-fold the maximum mitochondria branch length of DJ-1 KO cells, but not wild-type cells (p = 0.0248) (Fig. 3C and Supplementary Fig. 4). These results clearly demonstrate that 10 nM rotenone affects mitochondria in both genotypes, with a stronger effect on DJ-1 KO cells.

### Proteomics analysis of EV from WT and DJ-1 KO differentiated SH-SY5Y in oxidative stress reveals a specific DJ-1 dependent signature

**Fig. 4:**
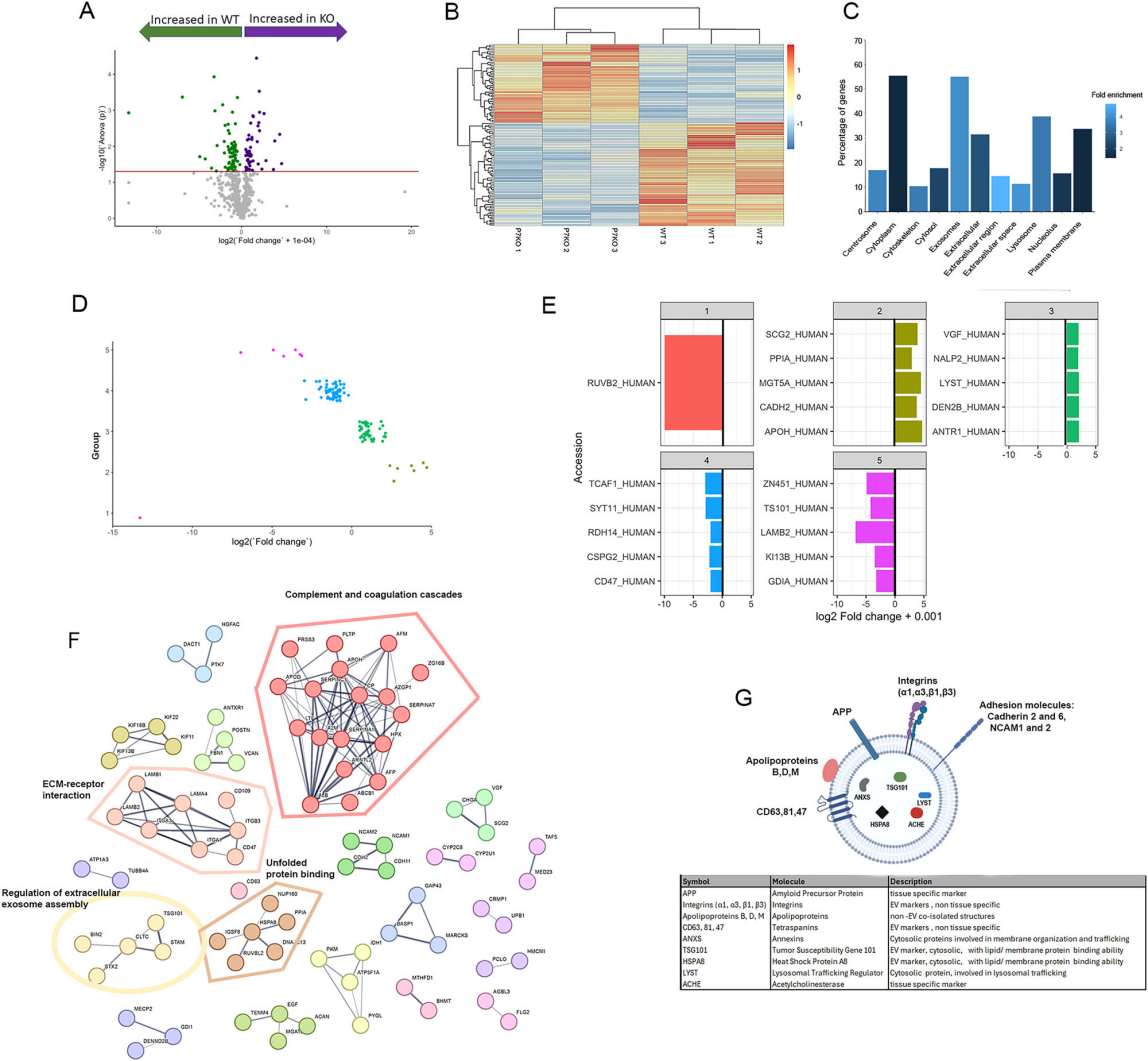
Mass spectrometry analysis reveals a DJ-1 dependent proteomic EV signature. **A** Volcano plot of protein fold changes in DJ-1 KO EV compared to WT EV upon rotenone treatment; log2 fold change +0.0001 (+0.0001 removes errors for proteins with quantity of 0 in DJ-1 KO) on x axis and -log10 p value on y axis; red line = p value threshold, proteins above line have p value < 0.05 and are significantly different. **B** Heatmap of differentially expressed proteins by unsupervised hierarchical clustering. Each column represents an individual EV sample, each row represents all the differentially expressed proteins. p value of less than 0.05. Samples and proteins are clustered based on euclidean distance. **C** Fold enrichment of cellular component terms in the total identified protein set. Y axis = percentage of genes (% of proteins in the total protein set with the specific associated term). Colour gradient = fold enrichment compared to Uniprot human protein dataset. The “Exosomes” term showed a substantially higher enrichment compared to the background than cytoplasm, with a fold enrichment of 3.9 vs 1.4. Furthermore, other significantly enriched terms linked to EV were present, including “Extracellular”, “Extracellular region”, “Extracellular space”, “Plasma membrane”, and “Lysosome” with fold enrichments of 2.5, 4.8, 4.1, 1.4, and 3.5 respectively. **D** Kmeans clustering of significantly different proteins by grouping on fold change. Significantly different proteins grouped by mini-batch kmeans algorithm with kmeans++ initializer; groups on y axis and log2 fold change on x axis; each point = 1 protein. Group 1, 1 protein absent in EV from DJ-1 KO; group 2, 7 proteins highly enriched in EV from DJ-1 KO; group 3, 43 proteins lowly enriched in EV from DJ-1 KO; group 4, 59 proteins lowly enriched in EV from WT; and group 6, 6 proteins highly enriched in EV from WT. Most EV proteins were either in group 3 or 4, which together represent 87.9% of significantly different EV proteins. **E** Top 5 proteins based on fold change ranked by log2 (fold change +0.001) for each kmeans defined protein group. Proteins are represented by their Accessions on the y axis and log2 (fold change + 0.001) on the x axis. Colours according to group colours as in (**D**). **F** STRING protein-protein interaction network analysis of EV proteins identified by mass spectrometry. Interaction network was built from the list of 116 significant proteins (*p* < 0.05). Confidence threshold = 0.4 and clustering performed with a MCL inflation parameter of 3 (disconnected nodes not shown). **G** Schematic of main EV components identified by mass spectrometry in this study (created with Biorender).

We next explored EV cargo upon 10 nM rotenone treatment by mass spectrometry analysis. Oxidative stress can affect EV cargo in three ways: addition or removal of specific cargo such as anti-oxidants; change in the concentration of cargo, or chemical modification of cargo (protein oxidations, post-translational modifications [33]). Analysis of the EV proteome from rotenone treated cells successfully identified 574 distinct proteins present in both WT and DJ-1 KO EV. Principal components analysis (PCA) showed a clear separation of WT and DJ-1 KO EV samples, primarily along PC1 and to a lesser extent along PC2 (Supplementary Fig. 5). Furthermore, out of the total 574 identified proteins, 116 or 20.2% possessed significantly different quantities (*P* < 0.05), and within this group, 50 were overexpressed in EV from DJ-1 KO and 66 in EV from WT (Fig. 4A and Supplementary Table 1). DJ-1 itself was not among the significant proteins. Notably, cellular compartment GO enrichment analysis against the Uniprot human database supported isolation and analysis of EV. Indeed, the “Exosomes” cellular compartment GO term was the most common term in the total dataset, along with “Cytoplasm” (55% of proteins, Fig. 4C).

**Fig. 5:**
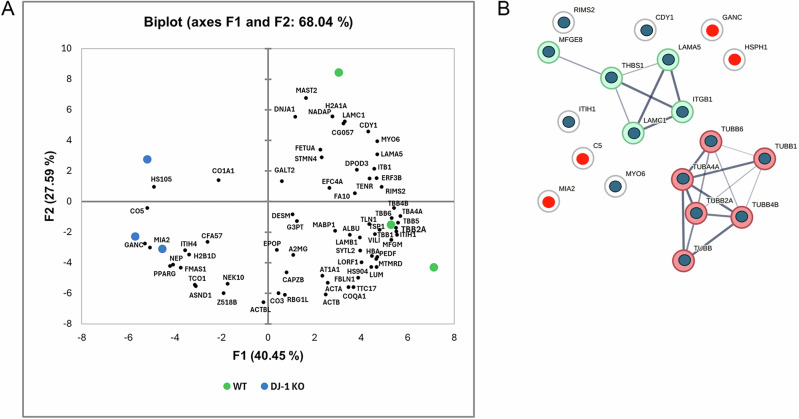
Label-free identification of protein oxidative modifications reveals a distinct signature in EV derived from DJ-1-deficient cells. **A** Loading plot of the first (PC1) and second (PC2) principal components derived from principal component analysis (PCA) of oxidative protein modifications detected in wild-type and DJ-1 KO EV following rotenone treatment. A total of 69 proteins were identified, among which 19 exhibited statistically significant differences between the two genotypes. **B** STRING protein-protein interaction network analysis of the 19 significant oxidative protein modifications identified in A. Confidence threshold = 0.4 and clustering performed with a MCL inflation parameter of 3 (all nodes shown). Up-regulated and down-regulated proteins in DJ-1 KO cells are represented by red and blue nodes, respectively.

K-means clustering of the significantly different proteins (increased in either EV from WT or DJ-1KO) resulted in 5 groups (Fig. 4D). GO analysis of biological process for the fold change derived protein groupings showed that group 1 was represented by a single protein, RUVBL2, involved in regulation of DNA transcription and repair, and histone and chromatin modification [34]. Group 2 highlighted the theme of blood coagulation [35, 36], and a secondary theme of synaptic regulation. Groups 3 and 4 were enriched in proteins involved in vesicle trafficking regulation [37, 38], protein localisation/transport [39] and immunoregulation, and group 5 showed the themes of cell adhesion and differentiation. These themes were confirmed by STRING analysis of the identified proteins (Fig. 4F). To link our results to DJ-1 role in the protection from oxidative stress, we next looked at oxidative modifications within the identified EV proteins. PCA revealed a clear separation between the wild-type and DJ-1 KO groups (Fig. 5A, Supplementary Table 2), indicating that DJ-1 plays a role in modulating cellular response to oxidative damage. Indeed, a strong correlation was found between specific proteins and either the wild-type phenotype (Fig. 5A, green) or the DJ-1KO phenotype (Fig. 5A, blue). For example, wild type EV showed oxidation of Alpha-2-HS-glycoprotein (FETUA) and tubulins, while DJ-1 KO EV result in oxidation of complement 5 and HSPH1 (HS105).

### EV effects on macrophage migration are dependent on DJ-1 and rotenone-induced oxidative stress in donor SH-SY5Y cells

**Fig. 6:**
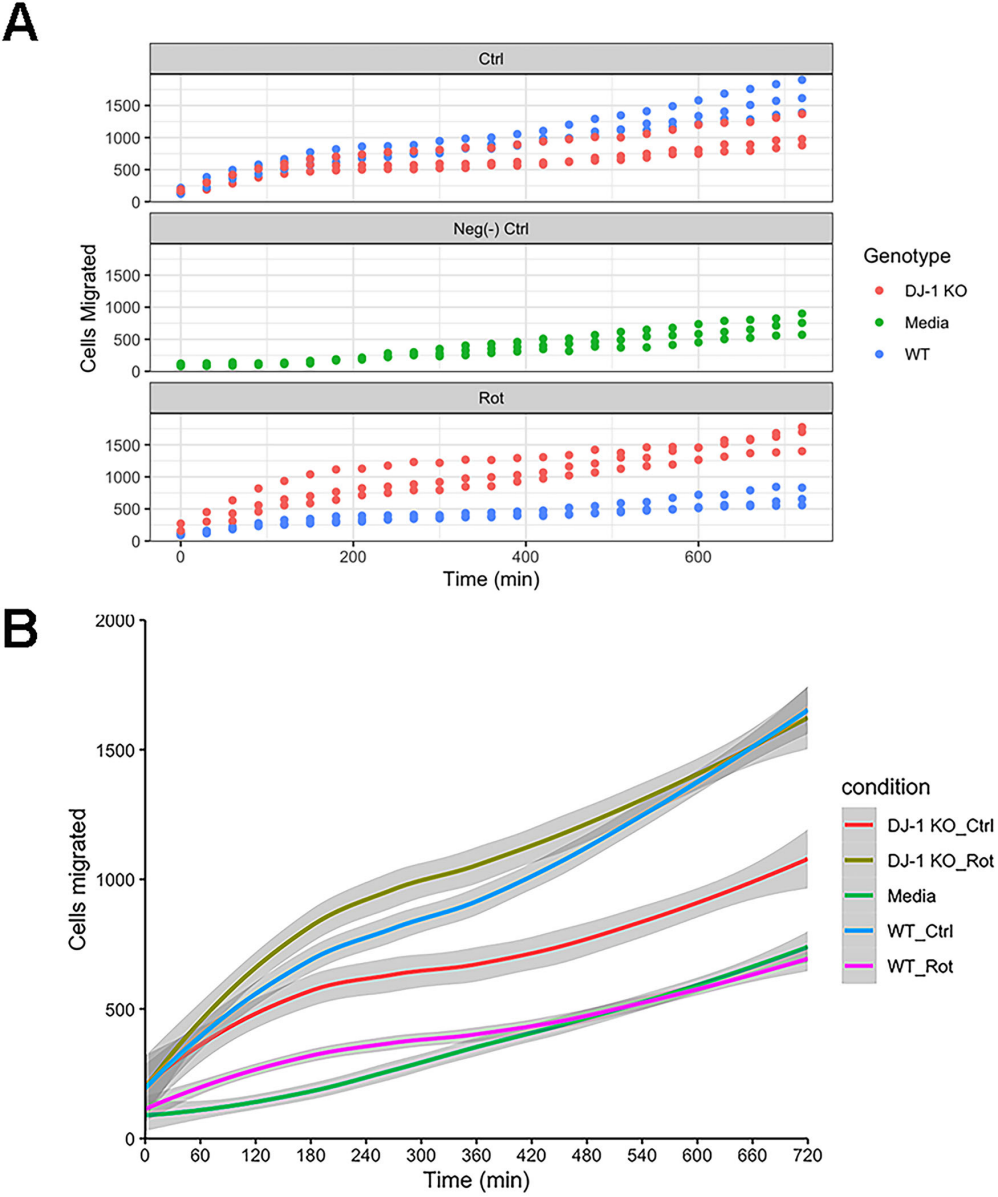
Promotion of macrophage migration by secretome is dependent on DJ-1 and rotenone-induced oxidative stress in source cells. **A** Plots of THP-1-derived macrophage migration promoted by secretome from WT and DJ-1 KO differentiated SH-SY5Y cells in control conditions and upon rotenone-induced oxidative stress. Top panel: secretome from healthy control cells, middle panel: negative control, bottom panel: secretome from rotenone treated cells n = 3. Differences in the trend line intercepts and overall rate of migration were assessed via a mixed effect model of the following form: Migrated cells ~ polynomial (Time, 2nd degree) ∗ condition +(1 + polynomial(Time, 2nd degree)|culture). Tukey HSD was used for multiple comparisons. **B** LOESS smoothing of migrated cell counts over time for each condition excluding positive control; error indicated by grey region surrounding lines.

As DJ-1 has been reported to modulate the activation of several immune cells including macrophages [40] and oxidation can modulate complement 5 ability to attract macrophages [41], we next investigated the ability of the secretome obtained from wild-type cells or DJ-1 KO cells to promote THP-1-derived macrophage migration (Fig. 6). We used the secretome instead of SEC purified EV to preserve the integrity of extracellular vesicle corona [42]. Interestingly, when comparing the effect of the secretome from wild-type and DJ-1 KO cells in control condition (Fig. 6A, B), we observed a stronger effect of wild-type cells compared to DJ-1 KO on THP-1 derived macrophages migration (p = 0.016), consistent with a decreased efficiency of EV mediated signal in the absence of DJ-1 in donor cells despite no change in EV number between the two genotypes (Fig. 2). However, when we compared the effect of the secretome from wild-type and DJ-1 KO cells upon rotenone treatment, a much stronger effect on THP1 migration was observed for DJ-1 KO cells than wild-type (p = 0.008). These results clearly indicate DJ-1 involvement in the modulation of macrophage migration via EV.

### DJ-1 KO alters EV response to rotenone in iPSC-derived neuronal cells

**Fig. 7:**
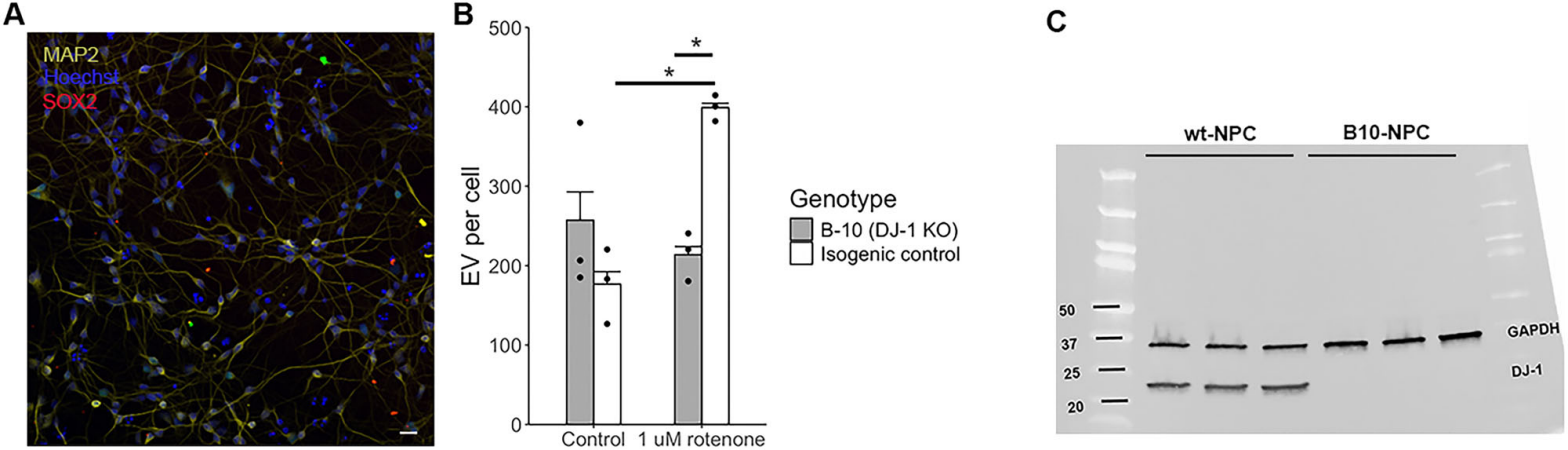
Rotenone treatment of iPSC-derived neurons reveals genotype dependent differences in the EV response. **A** Representative image of iPSC-derived neurons 12DIV captured using a Leica SP8 confocal microscope; with fluorescence labelling of nuclei (Hoechst 33342, blue), canonical neuronal marker MAP2 (yellow) and pluripotency marker SOX2 (red). Scale bar= 5 µm. **B** Number of EV per cell detected on a NanoFCM nanoanalyser system. Each point represents an independent differentiation, n = 3, Welch’s T-test, Bonferroni corrected for multiple comparisons. Column height = mean, error bars = standard error. * P < 0.05. **C** Representative DJ-1 immunoblot in lysates of neuronal precursors from isogenic control and B10 cells used for this experiment (10 µg of protein per lane).

To verify the relevance of our results in SH-SY5Y cells, the effect of rotenone on EV populations was also studied in iPSC-derived neuronal cells. Neuronal cells were differentiated from iPSC with a 1 bp deletion in the *PARK7* gene and their isogenic control (Fig. 7A). We first identified a suitable rotenone concentration, to account for differences in cell sensitivity to oxidative stress compared to SH-SY5Y cells. To this aim, iPSC-derived neurons were treated with varying concentrations of rotenone for 24 h and the number of necrotic cells was analysed as described for SH-SY5Y cells by using a membrane-impermeable fluorescent dye. Of the treatments tested, 1 µM rotenone appeared the most suitable both by visual observation of cultures and assessment of necrotic percentage as it had no significant effect on the death of DJ-1 KO iPSC-derived neurons (Supplementary Fig. 6). The number of EV per iPSC-derived neuron in untreated/treated DJ-1 KO and isogenic control cultures (MAP2 positive, SOX2 negative) was assessed via flow cytometry on a NanoFCM nanoanalyser system. In the absence of rotenone, the observed trend validated findings in SH-SY5Y cells, showing an increase in EV in DJ-1 KO neuronal compared to isogenic controls (Fig. 7B). The increase in EV upon rotenone treatment in the control cells was also confirmed (2.26-fold, p = 0.013, t-test, Bonferroni corrected). However, at 1 µM rotenone, isogenic control cells showed a significantly higher number of small EV than DJ-1 KO cells (1.86-fold, p = 0.035 t test, Bonferroni corrected), similar to what observed at 5 nM (not 10 nM) rotenone in SH-SY5Y cells (Fig. 2). These findings suggest that, despite different sensitivities to rotenone between iPSC-derived neuronal cells and SH-SY5Y cells, DJ-1 contributes to the regulation of EV-mediated response to oxidative stress.

## Discussion

EV increased production may represent a protective cell mechanism against oxidative stress: via their cargo, EV can stimulate pro-survival responses in recipient cells [43]. Alternatively, EV released in oxidative stress may exert a detrimental effect on recipient cells via their oxidized lipids and proteins cargo [44] though this may represent a mechanism by which a donor cell may seek “self-protection” through discard of oxidised components.

In this study, we observed an increase in EV upon rotenone treatment in both wild-type and DJ-1 KO cells, confirming the involvement of EV in the oxidative stress response. Strikingly, DJ-1 knockout cells required a higher concentration of rotenone to elicit an enhanced small EV response compared to wild-type cells (10 nM rotenone for DJ-1KO, 5 nM for WT). This supports the role of cellular DJ-1 as an oxidative stress sensor [29, 30], allowing wild-type cells to detect and respond to lower levels of oxidative stress more effectively than DJ-1 KO cells, and may explain why, in the absence of DJ-1, the EV response to mild oxidative stress was not observed.

At 10 nm, rotenone DJ-1 KO cells exhibited a marked increase in detectable EV. Interestingly, a much smaller but still significant difference in EV between the two genotypes was also observed in control condition at 48 h (Fig. 1). Thus, EV may represent a cellular mechanism for managing oxidative stress, which is already higher in DJ-1 KO cells compared to controls, even in the absence of rotenone. This is supported by our data on mitochondrial morphology and polarization state (Fig. 3).

Our proteomic data show that DJ-1 not only regulates the quantity of EV, but also influences the protein composition of EV under oxidative stress, indicating a DJ-1-dependent proteomic signature of EV upon oxidative stress. To link the proteomic signature to a functional readout, we proved that the secretome from cells lacking DJ-1 in oxidative stress condition has a dramatically different effect in stimulating macrophage migration. Macrophage migration models the responsive ability of the CNS resident macrophage population, microglial cells, and microglia-mediated neuroinflammation is a known player in the early stage of PD. Interestingly, knockout of DJ-1 in mice and primary microglia leads to an increase of the pro-inflammatory phenotype in microglia following LPS treatment, compared to controls [45, 46]. We observed that under control conditions, the secretome from wild-type cells showed a stronger ability to induce macrophage migration than the one obtained from DJ-1 KO cells. However, following rotenone treatment, the secretome from DJ-1 KO cells exhibited a significantly stronger effect than wild-type cells. These results suggest that a low level of oxidative stress can reduce cell migration. Indeed, in wild-type cells treated with 10 nM rotenone and in DJ-1 KO cells under control conditions (where oxidative stress is solely due to the absence of DJ-1), the secretome is less effective at promoting migration compared to the one from untreated wild-type cells. This could be easily explained by the loss of function of EV cargo molecules modified upon mild oxidative stress conditions. However, when both DJ-1 knockout and rotenone treatment (10 nM) are applied together, a much stronger effect in inducing cell migration was observed. The differences in migration observed indicate that the effect is not only linked to the number of EV produced, as 10 nM rotenone increases EV production in both genotypes, while enhanced migration occurs only when the secretome originate from DJ-1 KO cells. This suggests that the content of the EV, rather than their quantity, plays a key role in influencing migration. Furthermore, it implies that a certain threshold of oxidative damage may be necessary to activate an alternative signalling pathway, potentially related to the absence of DJ-1, which influences the observed cellular migration. In this context, the decrease in CD47 (group 4, fold change 0.242), in EV from DJ-1 KO cells compared to wild type, was of particular interest. CD47, a receptor belonging to the immunoglobulin superfamily, is constitutively expressed by neurons and provides “don’t eat me” signals to maintain microglia in a homeostatic state [47]. This down-regulation of CD47 might be responsible for the increased macrophage migration in absence of DJ-1. We also showed that DJ-1 clearly influences the oxidative profile of EV proteins, with C5 significantly more oxidized in EV from DJ-1 KO cells. Oxidized C5 within EV might contribute to neuroinflammatory processes by promoting microglial activation or astrocytic responses, thus exacerbating neurodegeneration [48, 49].

This study identifies a novel, EV-mediated pathway through which neuronal DJ-1 orchestrates the immune response to oxidative stress. We demonstrate that in the absence of DJ-1, oxidative stress triggers a functional switch in the protein content of EV, transforming them from homeostatic messengers into pro-inflammatory signals, able to powerfully increase macrophage recruitment. Our work thus identifies the DJ-1-dependent EV proteome as a key mediator of intercellular communication in oxidative stress. Therefore, elucidating the downstream effects of these EV on microglial phagocytosis and polarization represents a critical next step and a promising therapeutic axis to explore for PD pathogenesis.

## Methods

### SH-SY5Y cell culture

Wild-type SH-SY5Y were purchased from ATCC, product code ATCC-CRL-2266. *PARK7* (DJ-1) knock-out cell line was genetically engineered via CRISPR by Synthego (Redwood City, California) with a guide sequence of CAGGACAAAUGACCACAUCA. SH-SY5Y cells were grown in DMEM/ F-12 (1:1) Glutamax medium (Gibco, UK) supplemented with 10% v/v FBS (Gibco, UK), 100 units/ml penicillin (Gibco, UK), and 100 μg/ml streptomycin (Gibco, UK) in a 95% air/5% CO2 atmosphere. Validation of DJ-1 KO was performed by western blot on undifferentiated and differentiated cells (Supplementary Fig. 1A).

### THP-1 monocytes

Human THP-1 monocytes (ATCC; LGC Standards, Middlesex, UK; product code ATCC-TIB-202) were cultured in RPMI 1640 medium (Sigma Aldrich, UK) supplemented with 10% (v/v) FBS (Gibco, UK), 1% penicillin–streptomycin and 1% L-glutamine (Sigma Aldrich, UK) and incubated at 37 °C and 5% CO_2_. Fresh medium was added upon expansion to a cell density of 5 × 10^5^–1 × 10^6^/ml. For differentiation into macrophage-like cells, THP-1 monocytes were centrifuged at 300 × g for 5 min and resuspended in fresh complete RPMI 1640 medium at a density of 5 × 10^5^cells/ml before differentiation was stimulated with 100 nM dihydroxyvitamin D3 (VD3; Enzo Life Sciences, UK) and incubation at 37 °C for 48 h to allow for complete differentiation into macrophage-like cells.

### EV detection by flow cytometry

Growth medium was collected, centrifuged at 300 × *g* at 4 °C for 5 min to remove dead cells, and the supernatant was then centrifuged again at 2000 × *g* for 20 at 4 °C to remove cellular debris. EV in collected supernatant were stained overnight by 5 µM Bodipy FL-SE (Invitrogen, UK) or 1 h at room temperature with 40 nM Memglow (Universal Biologicals, UK). The following day EV concentration and sizes were analysed by flow cytometry using a Beckman Coulter Cytoflex S. The detectors employed were FITC and violet light side scatter (SSC_1). Megamix-Plus SSC and Megamix-Plus FSC standardisation beads (1:1 mix) (BioCytex, UK), ranging from 100 nm to 900 nm diameter were employed to generate EV gates. The acquisition settings were as follows: SSC_1 threshold of 18,000 and gain of 400; FITC gain of 250. The flow rate was set to 10 µl/min, and sample analysis stopped at 30,000 total fluorescent positive EV events detected. EV count was normalised to cell protein amount or cell number in each well, depending on the experiment.

### EV Cryo-EM and confocal microscopy

Three T-75 laminin coated flasks per genotype were seeded with 800,000 WT or DJ-1 KO SH-SY5Y cells in 16 ml DMEM F-12 glutamax medium supplemented with FBS and P/S. Cells were then differentiated and EV were collected as described above. EV supernatant was harvested and concentrated to 500 µl via centrifugation in Amicon 30 K centrifugal filter units at 3260 x g at 4 °C. EV were then purified via IZON qEV size exclusion chromatography columns according to the manufacturer’s instructions. Pure EV samples were then concentrated again via centrifugation in Amicon 30 K centrifugal filter units at 3260 x g at 4 °C from 3 ml to 200 µl and placed on ice.

Five microliters of each sample was applied to a freshly glow-discharged lacey carbon grid and plunge-frozen using a Leica GP2 plunge-freezer. Grids were imaged using a JEOL 2200 FS with a Gatan K2 camera.

For confocal microscopy, concentrated pure EV samples were stained with 2.5 mM Memglow^TM^ Green for 1 h at RT. 10 µl of EV sample was then imaged on a Leica SP8 Falcon confocal microscope using the Alexa 488 dye assistant settings.

**EV protein analysis by mass spectrometry** was performed as described in [50] (see Supplementary Information).

### Secretome collection for migration assay

SH-SY5Y cells were seeded at a density of 8 × 10^5^ cells in T75 flasks, differentiated and treated with 10 nM rotenone as described above. Cells were removed by centrifugation at 300 × *g* for 5 min at 4 °C, and the supernatant harvested. EV-containing supernatant was centrifuged at 2000 × *g* for 20 min at 4 °C to remove cellular debris, the supernatant harvested, and next concentrated to a volume of 700 µL using 30 kDa Amicon vertical centrifugal filter column at 4 °C. Migration assay was performed as described in the Supplementary Information.

## Supplementary information

Supplementary data

Supplementary Table 1

Supplementary table 2

Supplementary methods

uncropped DJ-1 and GAPDH iPSC derived neurons

uncropped DJ-1 diff WB

uncropped loading DJ-1 diff WB

uncropped DJ-1 undiff WB

uncropped loading DJ-1 undiff WB

## Data Availability

All data supporting the findings of this study are available in the Supplementary Material.
