# Supplementary material for: Parkinson’s associated protein DJ-1 regulates intercellular communication via extracellular vesicles in oxidative stress
Source: Cell Death Discov. 2025 Nov 21;11:539. doi: 10.1038/s41420-025-02845-7 (PMC12639138; doi:10.1038/s41420-025-02845-7)
Supplement: Supplementary file 1 — Supplementary data [file 41420_2025_2845_MOESM1_ESM.docx]

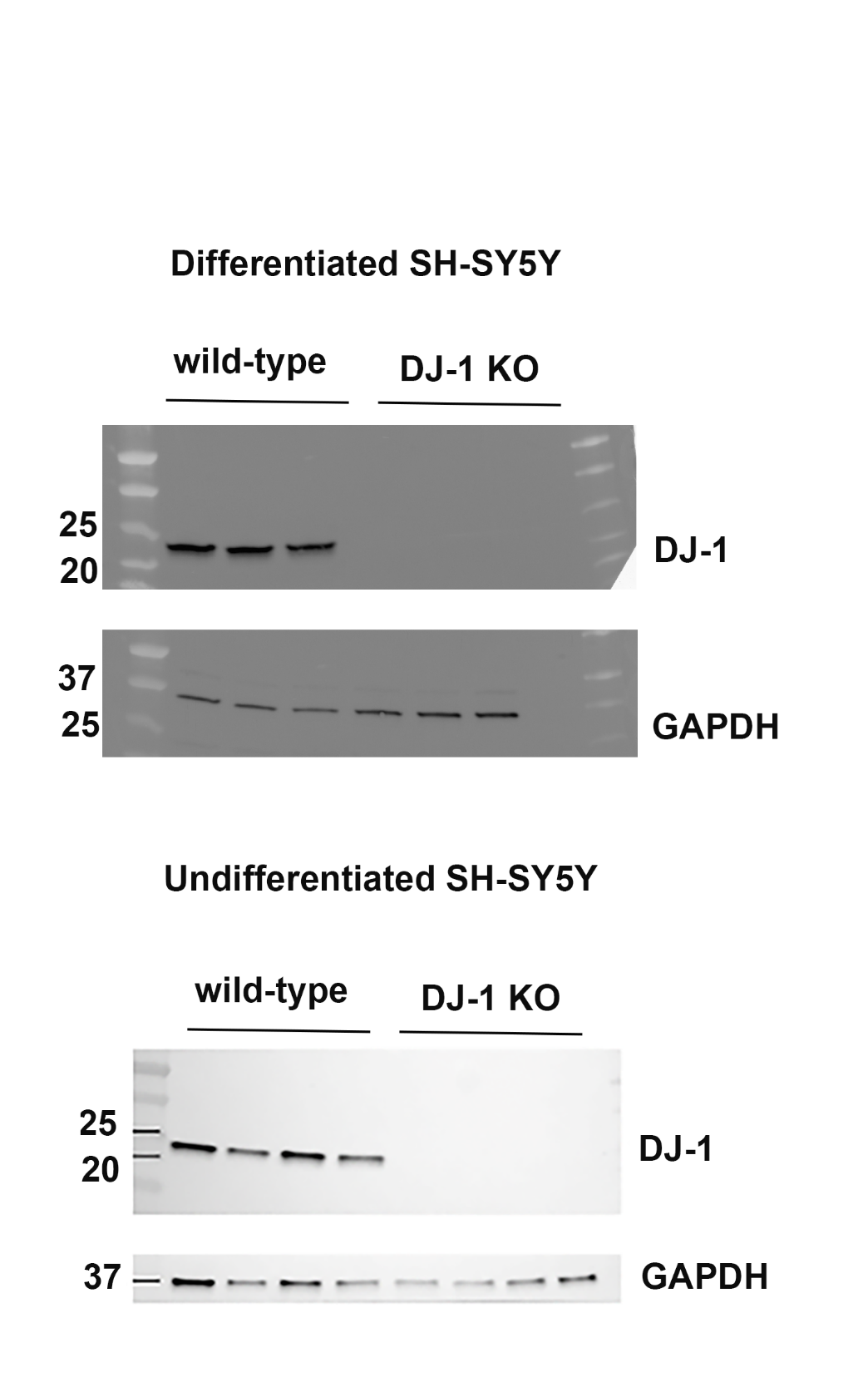


**Supplementary Figure 1A: Validation of DJ-1 KO in differentiated and undifferentiated SH-SY5Y cells.** Representative DJ-1 immunoblot in lysates of differentiated and undifferentiated SH-SY5Y cells (10µg of protein per lane). Loading control= GAPDH.

**
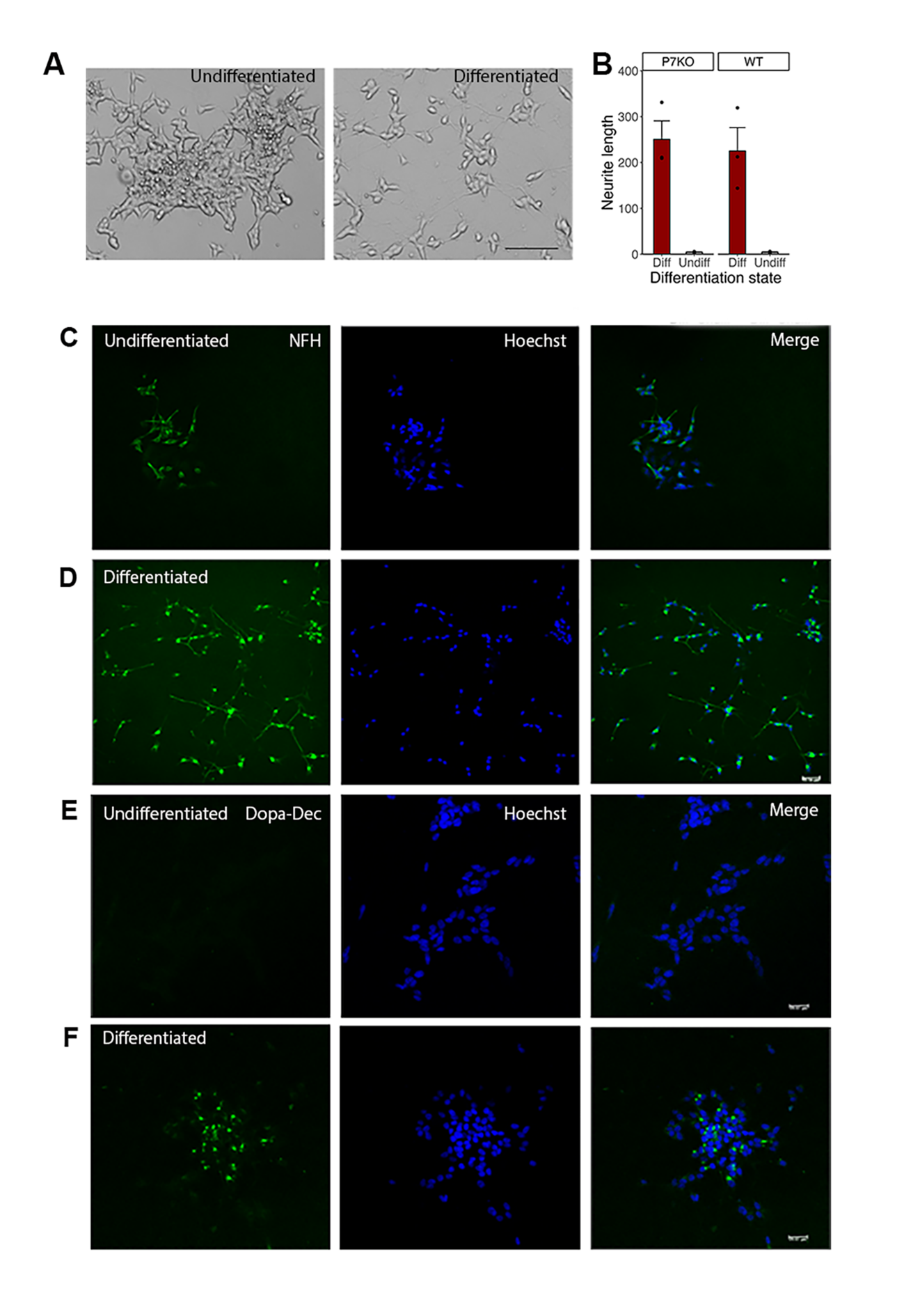
**

**Supplementary Figure 1B:** **Differentiation of SH-SY5Y results in enhanced neurite length and increased level of neuronal markers.**

A. Representative bright-field images of undifferentiated (left panel) and differentiated (right panel) SH-SY5Y cells, (10 X magnification on a Cytation 5 wide-field microscope), Scale bar = 100 µm. B. Neurite length quantification of undifferentiated and differentiated cells (mean+SD) n =3.

C-F: Confocal microscopy reveals changes in expression of NFH and DOPA-decarboxylase in SH-SY5Y upon differentiation. Scale bar = 55 µm in C and D and = 30 µm in E and F.


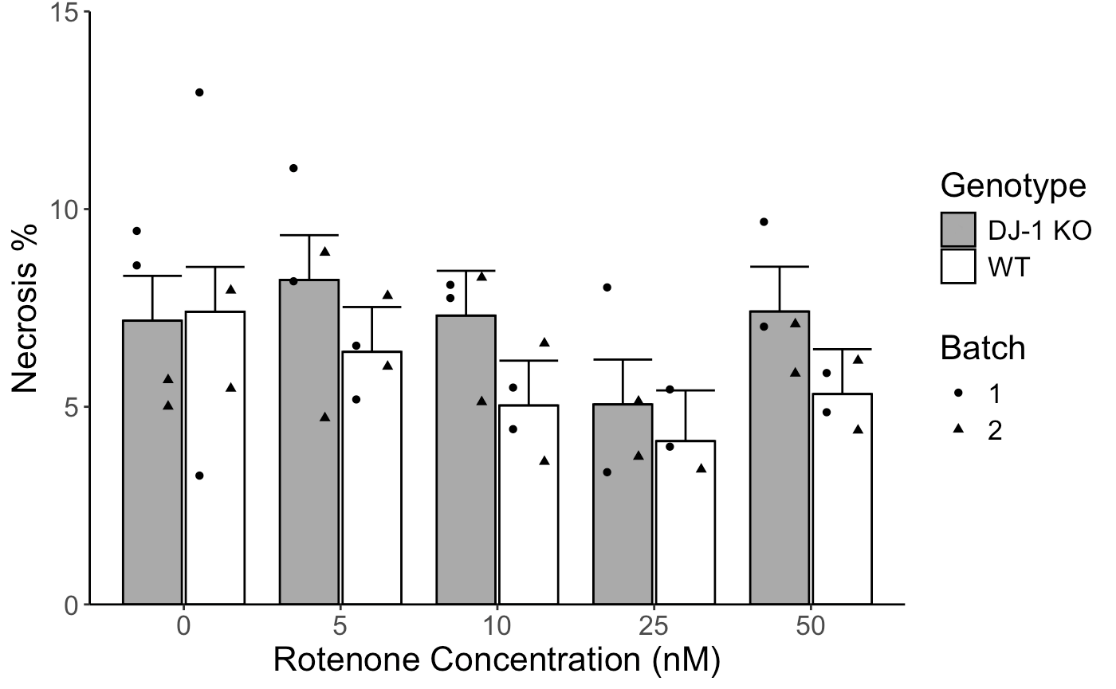


**A**

**
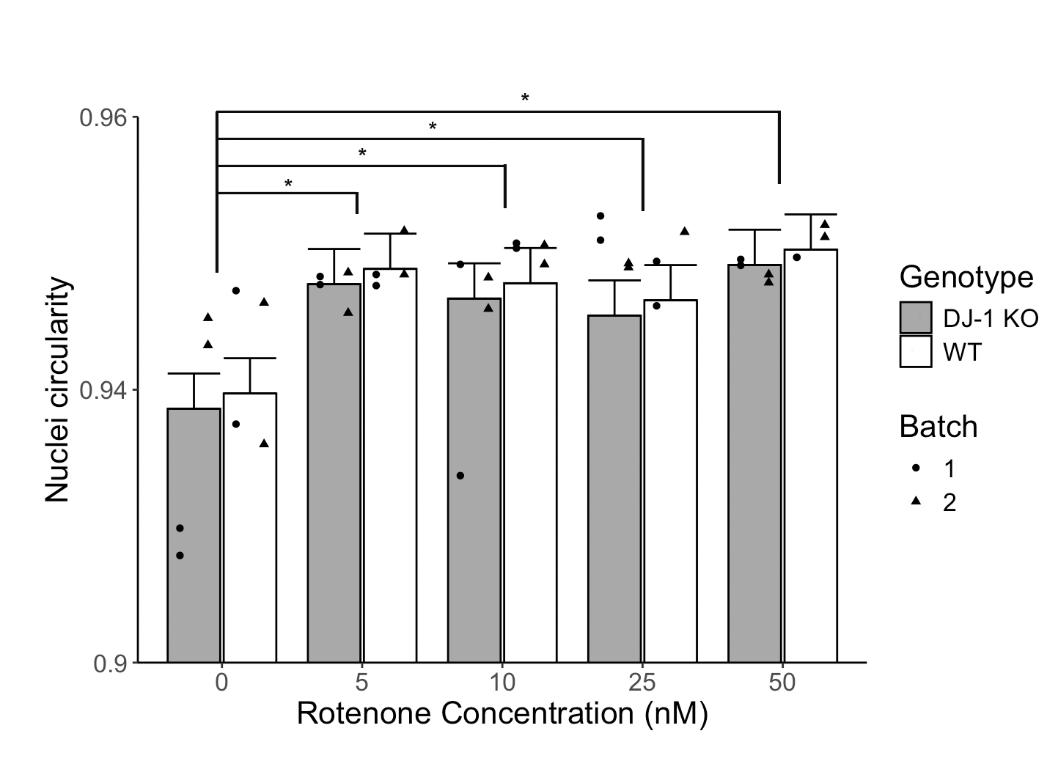
**

**B**

**Supplementary Figure 2: Rotenone treatment (24h) results in changes in nuclei circularity.**

A. No significant differences were detected in the percentage of necrotic nuclei upon rotenone treatment. B. Nuclei circularity increases at 5, 10, 25 and 50nM rotenone for both genotypes compared to their respective control. Bar height = least squares mean. Error bars = standard error. n = 4. Statistical analysis was performed in R via the emmeans package using pairwise t tests with fdr p value adjustment.

**

**

**Supplementary Figure 3: Effect of rotenone treatment (24h) on large EV.**

Large EV (>200 nm diameter) were detected on Cytoflex S system, EV size was assessed by violet-light side scatter. EV events were separated from noise by the fluorescence of Bodipy-FL-SE staining. Analyses of large EV number were carried out using the emmeans package in R on a mixed effect model of the form: EV number ~ Rotenone Concentration ∗ Genotype + (1|batch) using lme R package. n=9. Column height = least square mean calculated by the emmeans R package. Column height = least square mean calculated by the emmeans R package. Error bars = standard error. In contrast to the specific changes observed for small EV, rotenone treatment did not induce any significant changes in the release of large EV between the genotypes.


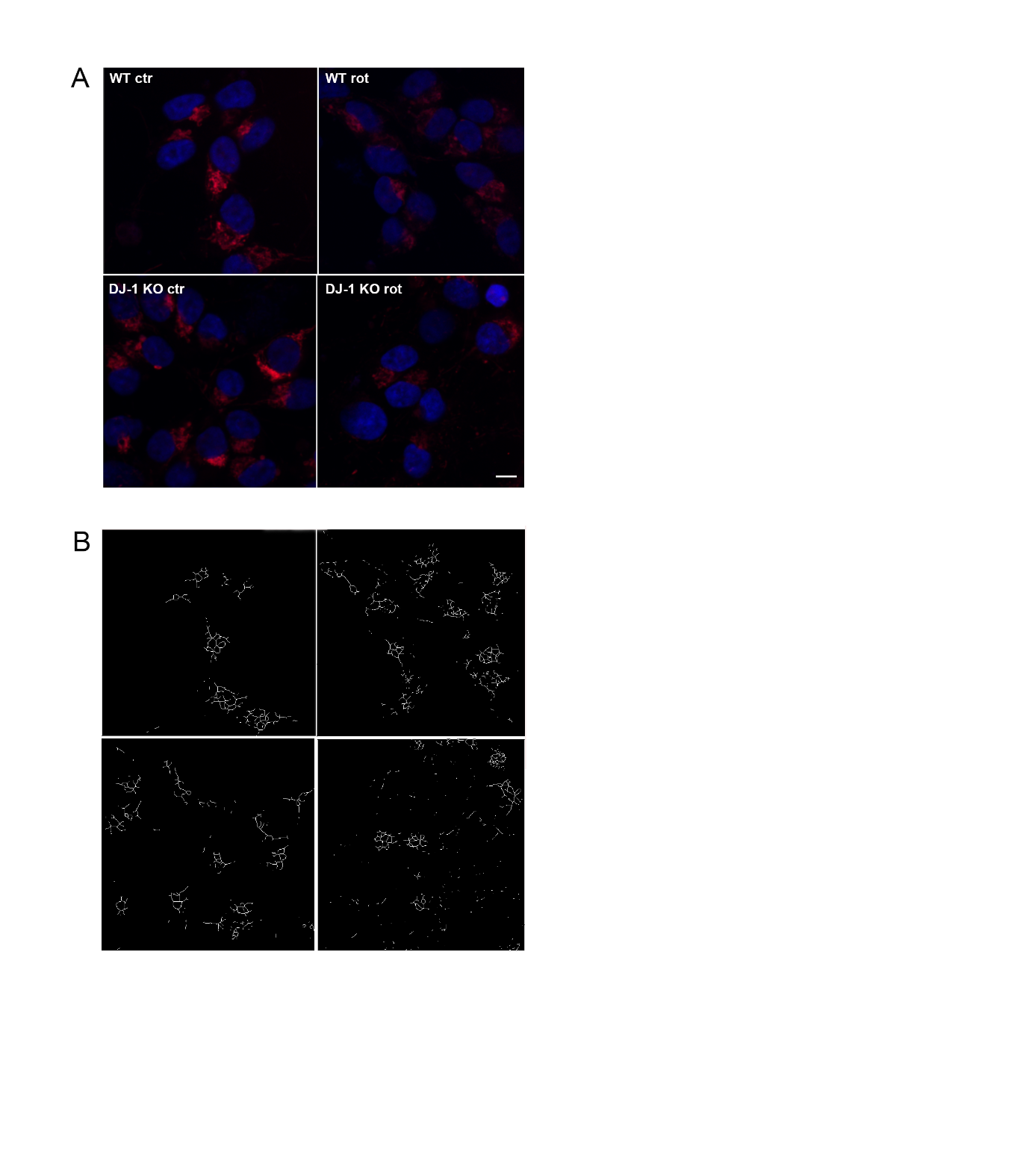


**Supplementary Figure 4**: **Effect of 10nm rotenone treatment (24h) on mitochondria.**

A. Representative images of mitochondria for each condition upon staining with MitoSpy Orange. Scale bar = 6.5µm. B. The skeletonized mitochondrial networks illustrate a disrupted and fragmented morphology in DJ-1 KO cells after rotenone treatment, characterized by fragmented and less interconnected branches. Images correspond to those in panel A and were generated in FIJI as described in Supplementary methods.


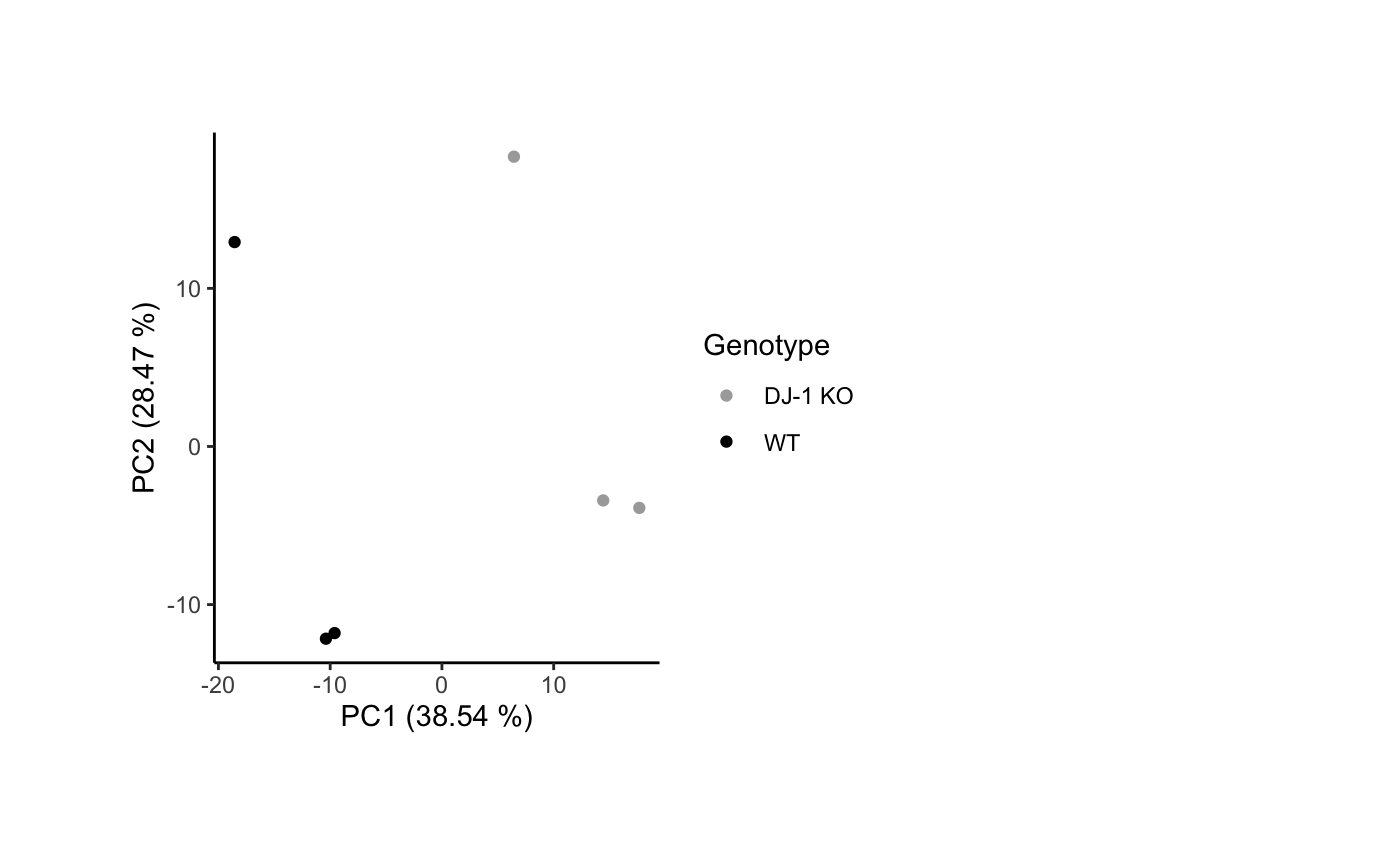


**Supplementary Figure 5:** Principal component analysis of the total identified protein dataset for WT and DJ-1 KO EV upon 10nM rotenone treatment.


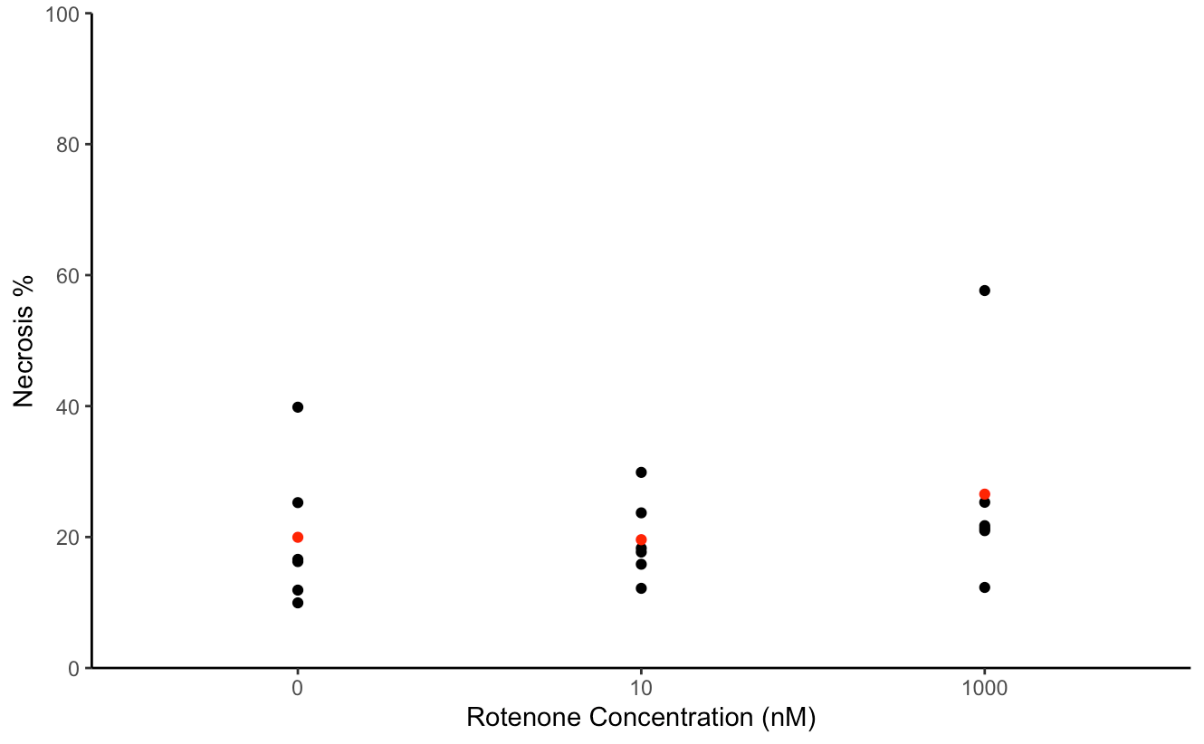


**Supplementary Figure 6**: Rotenone treatment (24h) does not induce necrosis in iPSC derived wild-type neuronal cells. Percentage of necrotic cell determined as in Supplementary figure 2 A. Each black dot = 1 culture. Red dot = mean.
