# Supplementary material for: Parkinson’s associated protein DJ-1 regulates intercellular communication via extracellular vesicles in oxidative stress
Source: Cell Death Discov. 2025 Nov 21;11:539. doi: 10.1038/s41420-025-02845-7 (PMC12639138; doi:10.1038/s41420-025-02845-7)
Supplement: Supplementary file 4 — Supplementary methods [file 41420_2025_2845_MOESM4_ESM.docx]

**Supplementary methods**

***SH-SY5Y differentiation and oxidative stress treatment***

Cells were plated on laminin (Corning, UK) coated (10 ug/ml) 6 well plates (1 × 10^5^ cells per well) for EV flow cytometry analysis, coverslips (1 × 10^5^ cells per well) for immunofluorescence studies or 12 well plate (5 × 10^4^ cells per well) for rotenone treatment. For differentiation, 48 h after plating cells were treated with 10 μM Retinoic acid (Sigma Aldrich, UK) in DMEM F-12 Glutamax supplemented with FBS and P/S for 5 days, followed by 5 days further treatment with 50 ng/ml BDNF (Peprotech 450-02) in FBS free medium, refreshed every 48h. Treatment of cells with rotenone (Sigma Aldrich, UK) was carried out from a 1000x stock solution in DMSO so that no more than 0.1 % DMSO was present in cell growth medium. Cells were treated with toxin concentrations ranging from 0-250 nM rotenone for 24 h.

***Cell viability analysis***

Following rotenone treatment, ReadyProbes^TM^ Cell viability kit (Invitrogen, UK) was used according to manufacturer’s instructions to evaluate blue (total) and green (necrotic) nuclei. Incubation was for 5 min at 37°C and 5 % CO2. After staining cells were imaged on a Cytation 5 microscope (BioTek) via an automated protocol using the fluorescent channel settings for DAPI and GFP. Each well was imaged at 9 regions of interest (ROI) evenly spaced around the well centre. Autofocus with scan occurred at each new image location in the DAPI channel. Counts of nuclei stained with ReadyProbe^TM^ blue (total) and ReadyProbe^TM^ green (necrotic), and analysis of nuclear morphology (size and shape) was performed in FIJI (ImageJ). Necrotic nuclei were counted via the same method as total nuclei counting and morphology analysis was undertaken by using the Stardist deep learning plugin for nuclei ROI creation (<https://github.com/stardist/stardist-imagej>) with the following parameters: model = “versatile (fluorescent nuclei)”; probability threshold = 0.4; allowed object overlap proportion = 0.4. The predictive model employed for necrotic nuclei percentage was as follows: 𝑑𝑒𝑝𝑒𝑛𝑑𝑎𝑛𝑡 𝑣𝑎𝑟𝑖𝑎𝑏𝑙𝑒 ~ 𝑅𝑜𝑡𝑒𝑛𝑜𝑛𝑒 𝐶𝑜𝑛𝑐𝑒𝑛𝑡𝑟𝑎𝑡𝑖𝑜𝑛 ∗ 𝐺𝑒𝑛𝑜𝑡𝑦𝑝𝑒 + (1|𝐵𝑎𝑡𝑐ℎ) +(𝐶𝑜𝑛𝑐𝑒𝑛𝑡𝑟𝑎𝑡𝑖𝑜𝑛|𝐵𝑎𝑡𝑐ℎ). For nuclei circularity and integrated density instead the model used was:

𝑑𝑒𝑝𝑒𝑛𝑑𝑎𝑛𝑡 𝑣𝑎𝑟𝑖a𝑏𝑙𝑒 ~ 𝐶𝑜𝑛𝑐𝑒𝑛𝑡𝑟𝑎𝑡𝑖𝑜𝑛 + 𝐺𝑒𝑛𝑜𝑡𝑦𝑝𝑒 + (1|𝐵𝑎𝑡𝑐ℎ). For the purposes of statistical

analysis toxin concentration was considered discrete. All mixed effects models were fitted via the

REML method.

***Immunostaining***

Cells were fixed in 4% w/v paraformaldehyde in PBS for 20 min and then incubated in 1% w/v bovine serum albumin (BSA) in PBS 0.2% v/v Triton (blocking solution) for 30 min at room temperature. Cells were then incubated with 1:1000 anti-DOPA-decarboxylase (mouse monoclonal; Abcam, ab211535) or 1:400 anti-NFH antibody (mouse monoclonal; Cell Signalling, mAb #2836), or anti-β-tubulin antibody (rabbit monoclonal; Cell Signalling, #2128) in blocking solution and incubated overnight at 4 °C. After washing in PBS, cells were incubated for 2 min in 1:2000 Hoechst 33342 trihydrochloride, 10 mg/ml solution (Invitrogen), in PBS. Incubation with the secondary antibodies (Alexa-488-conjugated goat anti-mouse Thermo Fisher, A11001; or Alexa-488-conjugated goat anti-rabbit (Thermo Fisher, A11034), for DOPA-decarboxylase, NFH and β-tubulin respectively, was in blocking buffer for 1 hr at room temperature. Finally, cells were rinsed in PBS and the final wash was replaced with Ibidi immersion oil (Ibidi, 50101) before imaging.

***Imaging***

For SH-SY5Y differentiation brightfield and fluorescence images were then taken of 7 different fields in each well on a Biotek Cytation 5 system (Agilent) at 20 X magnification. Neurite length was measured in a β-tubulin-stained field using the simple neurite tracer plugin in ImageJ (FIJI). n=3, with 3 wells analysed each time per condition. 100 cells were analysed per well (Supplementary Figure 1B).

***Mitochondria morphology analysis***

Differentiated SH-SY5Y cells were stained with 250 nM MitoSpy™ Orange CMTMRos (BioLegend 424804) for 30 min at 37 °C then washed once with PBS. Cells were then fixed with 4 % w/v paraformaldehyde and immunostaining was performed as described before using anti-ATP5-alpha primary antibody (Mouse monoclonal; Abcam, 14748) and Alexa-488-conjugated goat anti-mouse (Goat mono; Thermo fisher, A11001). Nuclei, ATP5-alpha and Mitospy orange were visualised on a Leica SP8 confocal microscope system at 20X and 40X magnification using the DAPI, Alexa 488 and Alexa 532 dye assistant settings with laser and detector settings kept consistent for all images of each marker. 10 images were taken per culture, with n = 4 spread evenly across 2 weeks of cultures, and for each condition at least 600 cells were imaged.

Mitochondrial morphology analysis was performed in FIJI (ImageJ) via an automatic analysis pipeline. Nuclei morphology was analysed using the StarDist (<https://github.com/stardist/stardist-imagej>) deep learning plugin for nuclei ROI creation with the following settings: modelChoice, Versatile (fluorescent nuclei); normalizeInput, true; percentileBottom, 1.0; percentileTop, 99.0; probThresh, 0.8; nmsThresh, 0.3; excludeBoundary, 2. Preprocessing was performed on all mitochondria stain images as follows: max intensity projection (Z project command); rolling ball background subtraction of radius 5; unsharp mask of radius 0.5 and mask 0.3; enhance local contrast with block size 199, histogram of 256, and maximum of 1.5; and finally median filter of radius 0.5. Fluorescence intensity was measured on a thresholded image of mitochondria with min and max pixel values of 40 and 255 and was normalised against nuclei counts.

Mitochondria branch morphology was analysed in ImageJ as follows: mitochondria selective staining was selected via Otsu auto thresholding, followed by skeletonisation using Skeletonize command, and skeleton analysis via the Analyze skeleton (2D/3D) command with prune set to none.

***Western blot***

Cells were washed twice with sterile PBS and then lysed on ice for 10 min in lysis buffer [17]. Lysates were centrifuged at 13,000 rpm for 10 min at 4 °C. Primary antibodies and dilutions were as follows: DJ-1 (rabbit polyclonal; Novus Biologicals; 1:2000), GAPDH (mouse monoclonal; Santa Cruz sc-265062; 1:500 dilution) and alpha-tubulin (mouse monoclonal, Cell Signaling Technology, #3873). Blots were developed using horseradish peroxidase (HRP)-conjugated secondary antibodies (1:10000; Vector Laboratories) and the ECL chemiluminescence system (SuperSignal West Dura Extended Duration Substrate, Thermo Scientific).

***EV isolation for mass spectrometry analysis***

Differentiated WT and DJ-1 KO SH-SY5Y cells were grown in T-75 flasks seeded at a density of 8x 10^5^ cells / flask. 3 EV samples per condition were prepared by pooling the growth media of 5 T-75 flasks per sample, spread equally across 3 weeks. EV were collected from the medium as described above, then the supernatant was concentrated to 500 μl via centrifugation in Amicon 30 K centrifugal filter units at 3260 x g at 4 °C. EV were then purified via size exclusion chromatography columns (qEV original, Izon) according to the manufacturer’s instructions. Purified EV fractions were then concentrated via centrifugation in Amicon 30 K centrifugal filter units at 3260 x g at 4 °C from 3 ml to ~ 100 μl and stored at -20°C prior use.

***Mass spectrometry analysis***

Extracellular vesicle (EV) samples purified by size exclusion chromatography (SEC) were analyzed for protein concentration using Bradford assay. Protein equivalents of 20 µg were denatured, reduced and alkylated in Laemmli buffer (Alfa Aesar, J61337) at 65 °C for 15 minutes. Reduced samples were then loaded and separated on a 10% SDS-PAGE. Resolved protein gels were stained overnight at 4 °C with Coomassie Brilliant Blue G-250 (0.5% w/v in 40% aqueous methanol and 10% glacial acetic acid). After destaining, each sample lane was divided into five uniform molecular weight band sections, providing five gel sections per sample. The sections were diced and transferred into polypropylene tubes (low protein binding; Eppendorf, Hamburg, Germany). In-gel digestion was performed according to the published protocol with slight modifications(1). Peptide extracts from each sample section were pooled into a single polypropylene tube, vacuum dried, and stored at −20 °C until mass spectrometry analysis.

For LC-MS analysis, the dried samples were reconstituted in 30 µL of 1% aqueous acetonitrile with 0.1% formic acid for liquid chromatography-tandem mass spectrometry (LC-MS/MS).

Peptide samples (5 µL) were injected onto a trap column (nanoEase M/Z Symmetry C18 Trap Column, 100Å, 5 µm, 180 µm × 20 mm, Waters, UK) on an nUPLC system (Acquity M Class, Waters, UK) operating in single-pump trapping mode at a flow rate of 5 µL/min using eluent B (acetonitrile in aqueous 0.1% formic acid). Peptides were separated at a flow rate of 0.5 µL/min on an analytical column (nanoEase M/Z ACQUITY UPLC BEH C18, 1.7 µm, 100 Å, 75 µm × 150 mm, Waters, UK) with the following gradient of eluent B: 0–45 minutes, 1–45% B; 45–49 minutes, 45–90% B; 49–52 minutes, 90% B; 52–67 minutes, 1% B. Peptide electrospray was formed at 2200 V using a PicoTip™ emitter (New Objective, Germany), and charged peptides were analyzed on a 5600 TripleTOF mass spectrometer (AB Sciex, Framingham, MA, USA) in information-dependent acquisition mode. The 10 most intense ions from each high-resolution MS survey scan were selected for high-sensitivity MS/MS, with a 30-second exclusion window for previously acquired peptide ions. The mass spectrometer was calibrated before acquisition to ensure high mass accuracy at both MS and MS/MS levels.

Relative protein quantification was performed using Progenesis QI for proteomics software (version 4.1, Nonlinear Dynamics, Newcastle, UK). Each sample sub-group (within the same molecular weight range) was aligned on a retention time vs *m*/*z* plot, ensuring the alignment of identical peptides across different samples. Data was further in-silico normalized (based on the peptide distribution with in-built Progenesis algorithm) to allow for the high-accuracy relative quantification. All sub-groups were combined using a multi-fraction setup to build up representative samples. Relative quantification included only protein-unique peptides. Merged data were exported as an .mgf file to Mascot search engine (Mascot Daemon platform, ver 2.5) which was searched against the curated SwissProt database with the following parameters: mass tolerance of 0.1 Da for MS and 0.5 Da for MS/MS spectra, a maximum of two trypsin missed-cleavages, *Homo Sapiens* taxonomy, and variable modifications of methionine oxidation and cysteine carbamidomethylation. To perform label-free identification of oxidative modification, the same data set was reanalysed to include variable modifications of methionine, lysine, proline, cysteine, tryptophan and histidine oxidation, cysteine di- and trioxidation, and lysine, cysteine and histidine modification via Michale addition of 4-hyroxy nonenal (HNE). Mascot searches were filtered to include peptides identified with minimum of 95% confidence including only scores with confirmed peptide identity (usually 31 for protein profiling, or 45 for oxidative modifications, or higher). Identified peptide list was exported back to Progenesis for the protein relative quantification between the samples. All keratins were treated as contamination and were excluded from the analysis. Exported protein relative quantification data tables were used for the quantitative gene ontology analysis using FunRich software tool.

***Bioinformatic analysis of the EV proteome***

Principal component analysis was carried out on EV samples using their protein quantity signatures to form the principal components via the R programming language. Kmeans clustering was carried out via the package ClusterR in the R programming language. The mini batch kmeans algorithm was employed with a kmeans ++ initialiser. Optimal centroids were decided by running kmeans algorithms with increasing cluster numbers until substantial diminishing returns in the decreasing “sum of squares” was observed. GO term enrichment and fold change compared to the Uniprot total *Homo sapiens* dataset and between analysed conditions was analysed via the FunRich software.

***THP-1 derived macrophage migration assay***

THP-1 monocytes were differentiated into macrophages via 48 hour treatment with 100 nM dihydroxy vitamin D3 at a cell density of 1x10^6^ cells/ml. 8x 10^4^ macrophages were subsequently seeded into each porous transwell insert in 300 µl serum-free RPMI medium, with each transwell situated in a well of 24 well Corning tissue culture companion plate containing 700 µl of secretome (derived from WT or DJ-1 KO differentiated SH-SY5Y in control or 10 nM rotenone treated conditions), or negative control (Serum-free RPMI). 8x10^4^ THP-1 derived macrophages were also seeded directly into wells in 700 µl serum-free RPMI without transwell insert as positive controls. Vertical migration of THP-1 derived macrophages was monitored on a Cytation 5 automatic microscope system. Positive controls were used to define the focal height employed for all imaging by focussing on the macrophages directly seeded into the wells and thus residing at the migration finish focal height. Bright-field images were captured at 4 X magnification for 12 h with 4 images being captured per well every 30 min. Each set of 4 images was subsequentially stitched together. A minimum pixel intensity threshold of 3000 was applied to generate a mask, holes in masks were filled and touching objects split. Cells were defined as objects with a diameter ranging from 10 to 50 µm, and cell counts generated. For this experiment n = 3 individual cultures. Differences in the trend line intercepts and overall rate of migration were assessed via a mixed effect model of the following form: Migrated cells ~ polynomial(Time, 2nd degree) ∗ condition +(1 + polynomial(Time, 2nd degree)|culture).

***iPSC derived neurons***

iPSC with a 1bp deletion in the *PARK7* gene and its isogenic control (A18945, DJ1 WT and A18945, DJ1 KO, 2B10 clone) were kindly provided by Dr Mark Cookson, NIH. iPSCs were maintained under a feeder-free condition with Essential 8 medium (Gibco) on 10 µg/mL vitronectin (Gibco)-coated plates. Cells were fed daily with full media changes and passaged using EDTA 0.5 mM (Lonza) at 80% confluence.

*Neuronal Induction:* After 24 hours from the plating, the medium was changed into neural induction media (NIM) composed of E6 (ThermoFisher), 2µM XAV-939 (HelloBio), 10µM SB431542 (HelloBio), 0.1µM LDN193189 (HelloBio). For cell seeding medium was supplemented with 10 µM RHO/ROCK pathway inhibitor Y-27632, and after 24 hours, this was removed. Cells were fed daily for 12 days. At around day 5, neural rosettes began to form.

*Neural Precursor Cells (NPCs) Differentiation*: Cells were detached using Accutase (Sigma Aldrich) at 37°C for 4 minutes. The cell suspension was then centrifuged at 200g for 5 minutes and the pellet resuspended in Neural Maintenance Media (NMM) composed of Advanced DMEM: F12 (ThermoFisher), Neurobasal Media (ThermoFisher), 1% Glutamax (ThermoFisher), 0.5X N2 (ThermoFisher), 0.5X B27 (ThermoFisher) and βMercaptoethanol (1:1000, ThermoFisher). Cells were seeded at a density of 100,000 cells/cm^2^ on 6-well plates or coverslips to perform immunostaining, coated with 20 µg/mL of Poly-L-Ornithine (ThermoFisher) and 10 µg/mL of laminin (Biolamina, LN511-0502). For cell seeding medium was supplemented with 10 µM Rock-Inhibitor, and after 24 hours, this was removed. Cells were then fed every 3-4 days and passaged at around 90% confluence. On day 7, FGF2 (1:10000, Qkine) was added to the medium.

*Neural Differentiation:* At passage 2 or 3, NPCs were transferred to the final plating format. They were seeded at 285,000 cells/well on coverslips in 24-well plates coated with 20 µg/mL of Poly-L-Ornithine (ThermoFisher) and 10 µg/mL of laminin (Biolamina, LN511-0502). Cells were seeded into NMM with 10µM Rock inhibitor. The day after, the media was changed into BrainPhys, SM1 (Stem Cell Technologies), 20ng/mL BDNF (Qkine), 20ng/mL GDNF (Qkine), 2µM Compound E (Stem Cell Technologies). Every 3 days, half media change was performed. After 6 days in the presence of Compound E, this was removed, and cells were fed every 3 days with BrainPhys, SM1, 20ng/mL BDNF and 20ng/mL GDNF. At DIV12, cells were used for the experimental procedures.

***Rotenone treatment***

At DIV12 iPSC-derived neuronal cells were treated with 1μM of Rotenone in DMSO for 24 hours and then fixed as previously described for immunofluorescence analysis.

***EV from iPSC-derived neurons***

Before fixation, the media was collected to isolate EV. EV concentration and size were analysed by nano flow cytometry using a NanoFCM Nanoanalyser system. The detectors employed were green fluorescence and violet light side scatter, and the system was set up according to manufacturer’s instructions. EV count was normalised to true nuclei count in each condition assessed by total culture growth area Tilescan, capturing images using the DAPI dye assistant settings of a Leica SP8 confocal microscope of the entire culture area and quantification of nuclei in FIJI (ImageJ).

1. Shevchenko A, Tomas H, Havli J, Olsen JV, Mann M. In-gel digestion for mass spectrometric characterization of proteins and proteomes. Nature Protocols. 2006;1(6):2856-60.
