# Supplementary figures and images for: Parkinson’s associated protein DJ-1 regulates intercellular communication via extracellular vesicles in oxidative stress
Source: Cell Death Discov. 2025 Nov 21;11:539. doi: 10.1038/s41420-025-02845-7 (PMC12639138; doi:10.1038/s41420-025-02845-7)

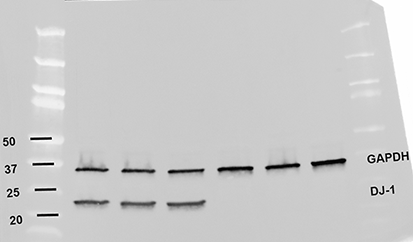

Supplement: Supplementary file 5 — uncropped DJ-1 and GAPDH iPSC derived neurons [file 41420_2025_2845_MOESM5_ESM.tif]

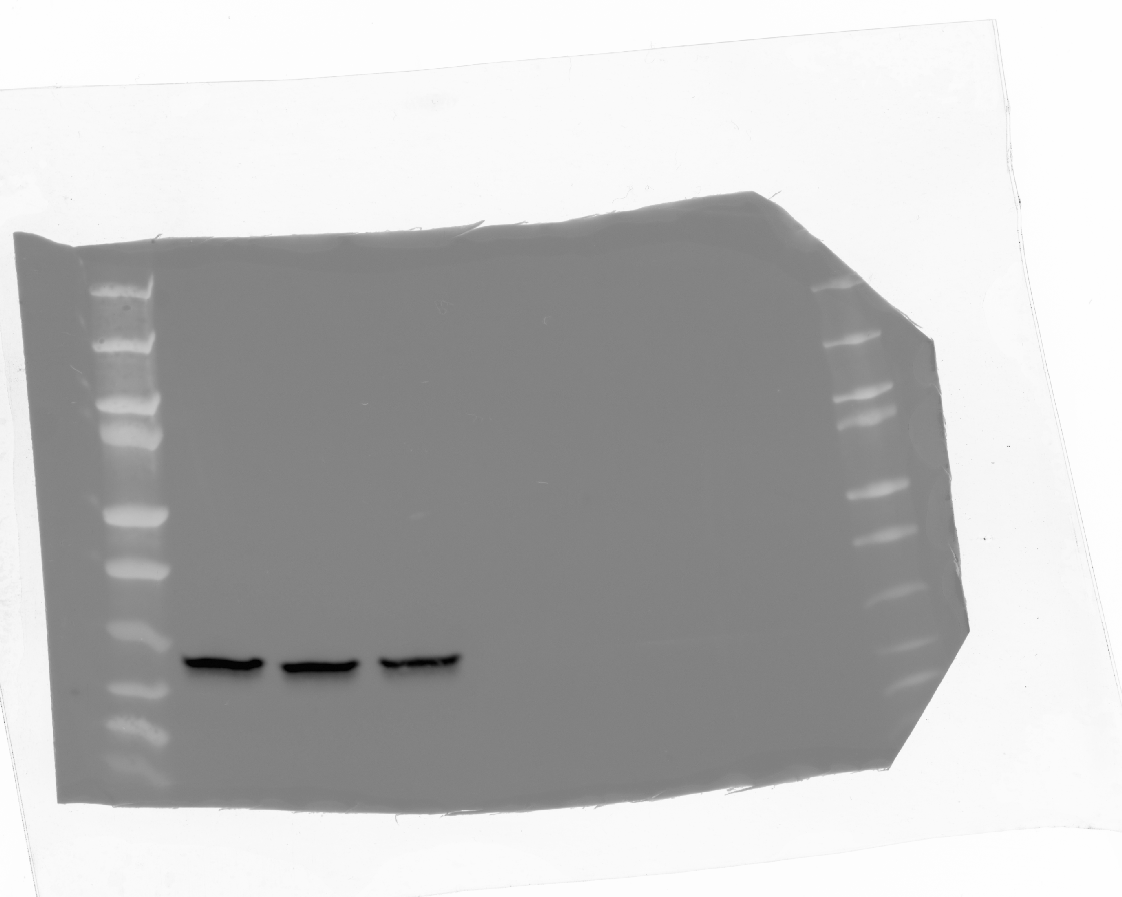

Supplement: Supplementary file 6 — uncropped DJ-1 diff WB [file 41420_2025_2845_MOESM6_ESM.tif]

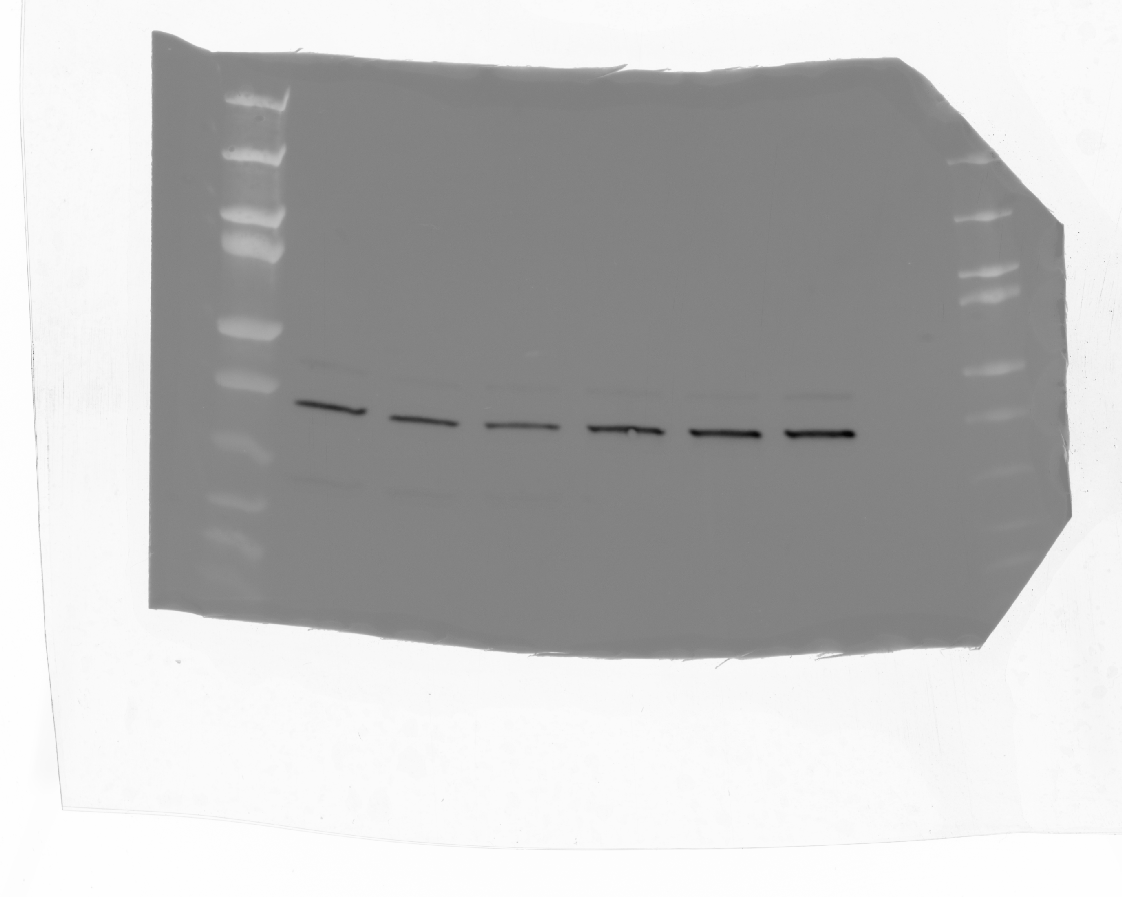

Supplement: Supplementary file 7 — uncropped loading DJ-1 diff WB [file 41420_2025_2845_MOESM7_ESM.tif]

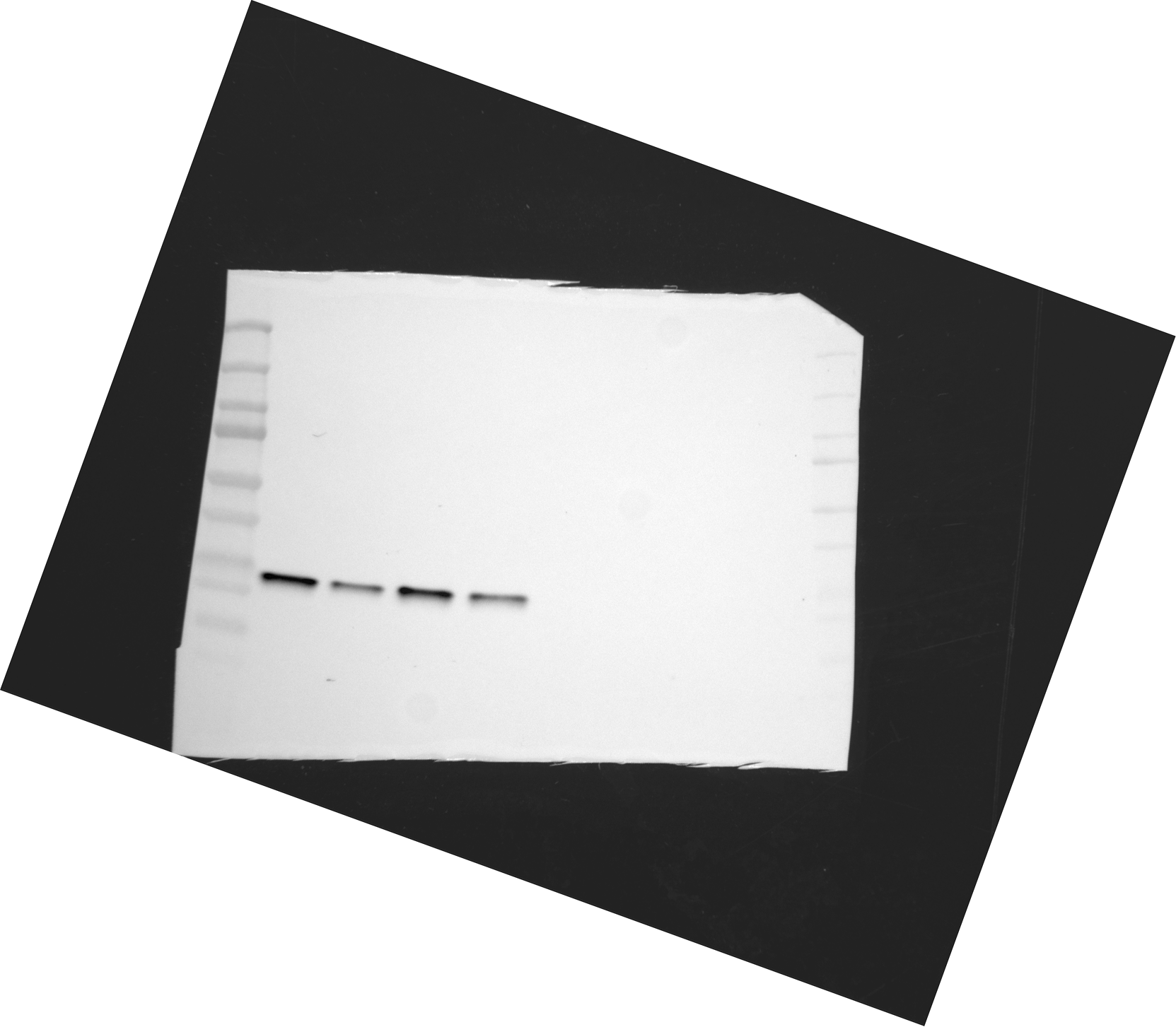

Supplement: Supplementary file 8 — uncropped DJ-1 undiff WB [file 41420_2025_2845_MOESM8_ESM.tif]

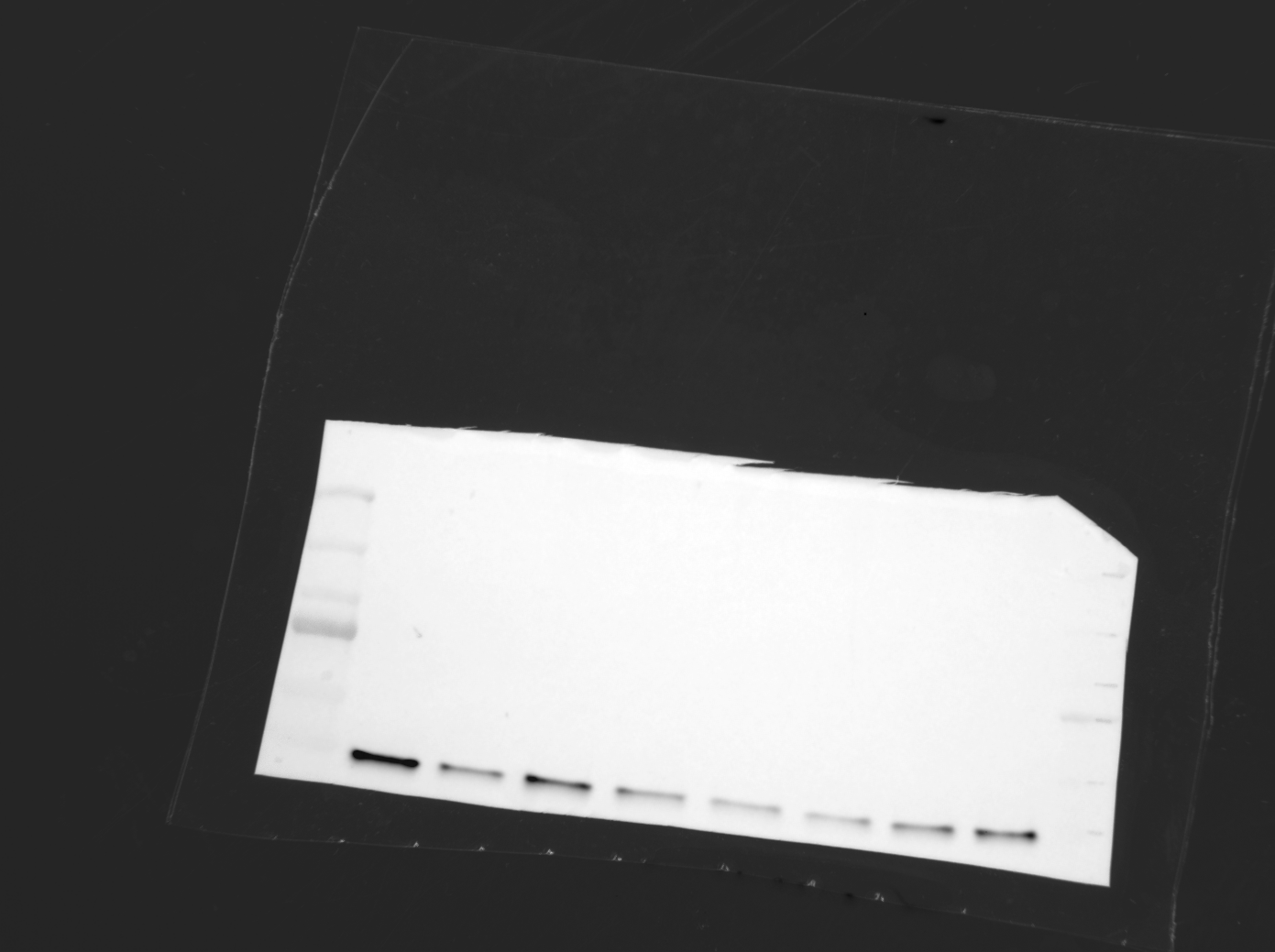

Supplement: Supplementary file 9 — uncropped loading DJ-1 undiff WB [file 41420_2025_2845_MOESM9_ESM.tif]
